# Prophylactic efficacy of an intranasal spray with 2 synergetic antibodies neutralizing Omicron

**DOI:** 10.1172/jci.insight.171034

**Published:** 2024-04-08

**Authors:** Xinghai Zhang, Feiyang Luo, Huajun Zhang, Hangtian Guo, Junhui Zhou, Tingting Li, Shaohong Chen, Shuyi Song, Meiying Shen, Yan Wu, Yan Gao, Xiaojian Han, Yingming Wang, Chao Hu, Xiaodong Zhao, Huilin Guo, Dazhi Zhang, Yuchi Lu, Wei Wang, Kai Wang, Ni Tang, Tengchuan Jin, Menglu Ding, Shuhui Luo, Cuicui Lin, Tingting Lu, Bingxia Lu, Yang Tian, Chengyong Yang, Guofeng Cheng, Haitao Yang, Aishun Jin, Xiaoyun Ji, Rui Gong, Sandra Chiu, Ailong Huang

**Affiliations:** 1 CAS Key Laboratory of Special Pathogens and Biosafety, Wuhan Institute of Virology, Center for Biosafety Mega-Science, Chinese Academy of Sciences, Wuhan, Hubei, China.; 2 Department of Immunology, College of Basic Medicine, and; 3 Chongqing Key Laboratory of Basic and Translational Research of Tumor Immunology, Chongqing Medical University, Chongqing, China.; 4 The State Key Laboratory of Pharmaceutical Biotechnology, School of Life Sciences, Institute of Viruses and Infectious Diseases, Chemistry and Biomedicine Innovation Center (ChemBIC), Institute of Artificial Intelligence Biomedicine, Nanjing University, Nanjing, China.; 5 Shanghai Institute for Advanced Immunochemical Studies and School of Life Science and Technology, Shanghai Tech University, Shanghai, China.; 6 University of Chinese Academy of Sciences, Beijing, China.; 7 Department of Endocrine Breast Surgery, The First Affiliated Hospital of Chongqing Medical University, Chongqing, China.; 8 Shanghai Clinical Research and Trial Center, Shanghai, China.; 9 The Second Affiliated Hospital,; 10 Institute of Life Sciences, and; 11 Key Laboratory of Molecular Biology for Infectious Diseases (Ministry of Education), Institute for Viral Hepatitis, Department of Infectious Diseases, The Second Affiliated Hospital, Chongqing Medical University, Chongqing, China.; 12 Division of Life Sciences and Medicine, University of Science and Technology of China, Hefei, Anhui, China.; 13 Mindao Haoyue Co., Ltd., Chongqing, China.; 14 Engineering Research Center of Protein and Peptide Medicine, Ministry of Education, Nanjing, China.

**Keywords:** COVID-19, Immunology, Drug therapy, Immunoglobulins, Structural biology

## Abstract

**BACKGROUND:**

As Omicron is prompted to replicate in the upper airway, neutralizing antibodies (NAbs) delivered through inhalation might inhibit early-stage infection in the respiratory tract. Thus, elucidating the prophylactic efficacy of NAbs via nasal spray addresses an important clinical need.

**METHODS:**

The applicable potential of a nasal spray cocktail containing 2 NAbs was characterized by testing its neutralizing potency, synergetic neutralizing mechanism, emergency protective and therapeutic efficacy in a hamster model, and pharmacokinetics/pharmacodynamic (PK/PD) in human nasal cavity.

**RESULTS:**

The 2 NAbs displayed broad neutralizing efficacy against Omicron, and they could structurally compensate each other in blocking the Spike-ACE2 interaction. When administrated through the intranasal mucosal route, this cocktail demonstrated profound efficacy in the emergency prevention in hamsters challenged with authentic Omicron BA.1. The investigator-initiated trial in healthy volunteers confirmed the safety and the PK/PD of the NAb cocktail delivered via nasal spray. Nasal samples from the participants receiving 4 administrations over a course of 16 hours demonstrated potent neutralization against Omicron BA.5 in an ex vivo pseudovirus neutralization assay.

**CONCLUSION:**

These results demonstrate that the NAb cocktail nasal spray provides a good basis for clinical prophylactic efficacy against Omicron infections.

**TRIAL REGISTRATION:**

www.chictr.org.cn, ChiCTR2200066525.

**FUNDING:**

The National Science and Technology Major Project (2017ZX10202203), the National Key Research and Development Program of China (2018YFA0507100), Guangzhou National Laboratory (SRPG22-015), Lingang Laboratory (LG202101-01-07), Science and Technology Commission of Shanghai Municipality (YDZX20213100001556), and the Emergency Project from the Science & Technology Commission of Chongqing (cstc2021jscx-fyzxX0001).

## Abstract 2

An intranasal spray containing a cocktail of two synergetic broadly neutralizing antibodies shows efficacy against Omicron and was found to be safe in healthy volunteers.

## Graphical abstract

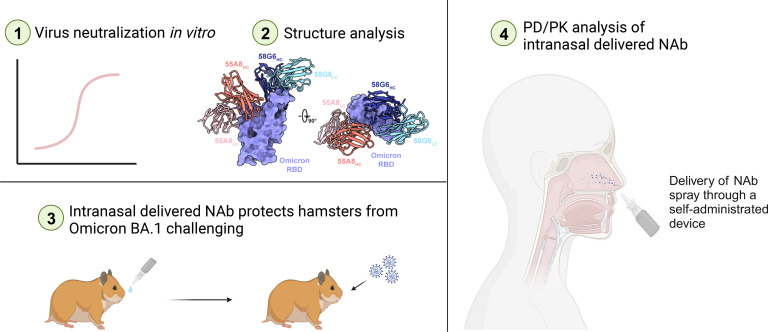

## Introduction

Since the outburst of COVID-19 in December 2019, SARS-CoV-2 continues to evolve substantially, acquiring sets of mutations with enhanced transmissibility, infectivity, and the ability to escape natural and acquired immunity (1–3). In contrast to other variants that preferentially engage in the lungs, Omicron alters the route of viral entry into host cells and is prompted to replicate in the upper airway (4–6). This causes more asymptomatic infections, contributing to silent spread of the viruses, and poses substantial difficulties in effective prevention of the spread of infection (7–9). Omicron and its subvariants exhibit a dramatic antigen shift, which has been shown to render the booster vaccination or recovery sera ineffective and cause breakthrough infections (10–13). Recent studies further hampered the active immune protection with evidences of inadequate protection against Omicron sublineages from vaccinated boosters based on Omicron BA.1 (14–16). Moreover, Paxlovid, a combination of 2 small molecule inhibitors that recently received emergency authorization for patients at higher risk of critical illness, failed to prevent close-contact infection in family members living with patients (17–19). Thus, alternative and supplemental prophylactic drugs are now urgently needed to prevent Omicron infection and subsequently block its transmission in the community (20–22).

Passive antibody administration based on NAbs has demonstrated protective efficacy in susceptible individuals who are moderately to severely immunocompromised or who have contraindication to vaccines, with promising potential in breaking the transmission chain (23–25). However, the high cost and the inconvenient intramuscular or i.v. way of administrations have drastically limited its application (20, 26, 27). To overcome these practical drawbacks, the passive transfer of inhibitors through the intranasal mucosal route may be a promising approach to prevent the spread of Omicron (28–31). Antibody delivery through an inhalant has been shown to facilitate early-stage contact with the pathogen in the respiratory tract (32–34). This may serve as an applicable platform enabling NAb enrichment in the route of viral entry (e.g., nasal cavity and the upper airway), overcoming the low and unsatisfying distribution of NAbs administered through a systemic route (35–37).

Here, we identified an intranasal-applicable NAb cocktail with broad neutralizing capability against Omicron and its sublineages, and we determined its synergetic neutralizing mechanism. We further investigated the prophylactic and therapeutic efficacy of this cocktail against authentic Omicron BA.1 in a hamster model, and we evaluated its safety and potential capability to block the infection of BA.5 via self-administrated nasal spray in an investigator-initiated trial.

## Results

### Broad neutralizing capability of the cocktail containing 58G6 and 55A8.

**Figure 1:**
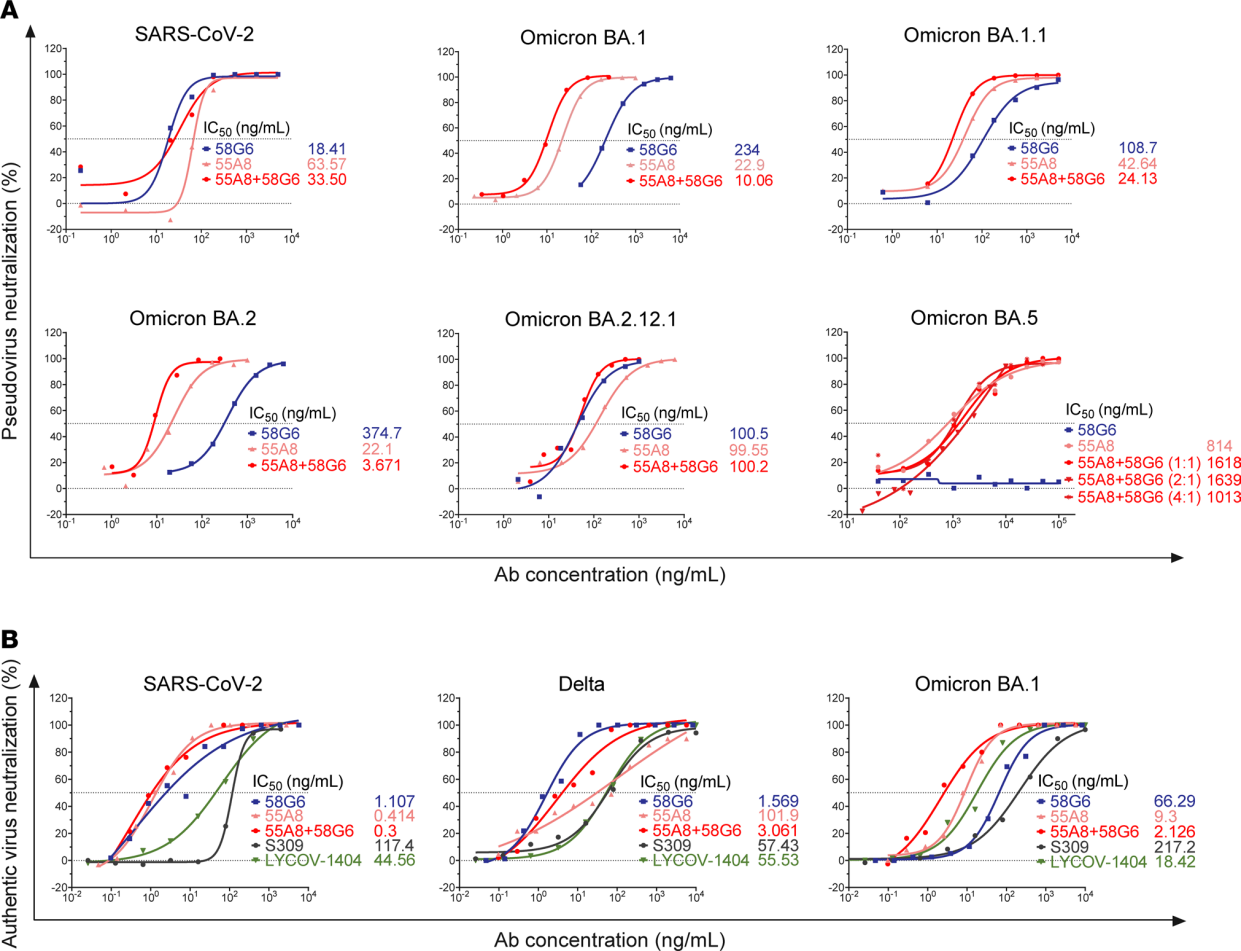
The cocktail of 58G6 and 55A8 broadly neutralized Omicron emerging variants. (**A**) The neutralizing potencies of 58G6, 55A8, and the cocktail of 58G6 and 55A8 against SARS-CoV-2, Omicron BA.1, BA.1.1, BA.2, BA.2.12.1, and BA.5 variants were measured with a pseudovirus neutralization assay. The dashed lines indicate a 0% or 50% reduction in viral neutralization. Data are representative of at least 2 independent experiments. (**B**) Neutralization against authentic SARS-CoV-2, Delta, and Omicron BA.1 viruses. The ratio of 55A8/58G6 is 1:1 in all experiments, unless otherwise marked.

58G6 and 55A8 were 2 NAbs identified from patients convalescing from COVID-19 in early 2019 (31, 38–40). 55A8 could bind to the spike (S) proteins of WT SARS-CoV-2 and its mutational variants, including Delta and Omicron BA.1, BA.2, and BA.5, as tested by enzyme-linked immunosorbent assay (ELISA) (Supplemental Figure 1, A and B; supplemental material available online with this article; https://doi.org/10.1172/jci.insight.171034DS1). Biolayer interferometry (BLI) analysis revealed that 55A8 exhibited strong binding affinities to the S proteins of SARS-CoV-2, Delta, and Omicron BA.5, at subpicomolar levels (<1 × 10^–12^ M) particularly for Omicron BA.1 and BA.2 (Supplemental Figure 1C). Furthermore, we confirmed that the cocktail of 55A8 and 58G6 could neutralize the pseudoviruses of multiple SARS-CoV-2 variant strains (Figure 1A and Supplemental Figure 2, A and B). Interestingly, the cocktail of 58G6 and 55A8 demonstrated evident synergetic effects against pseudotyped SARS-CoV-2 variants as well as the authentic Omicron BA.1, with the half inhibition concentration (IC_50_) exponentially lower than the currently approved NAbs for the emergency treatment of COVID-19 (Figure 1, A and B). However, we did not observe a synergetic neutralizing effect of this NAb cocktail against Omicron BA.5; the inclusion of 58G6 exhibited a lower neutralizing effect compared with that of 55A8 when used alone (Figure 1A). These results show that this cocktail consisting of neutralizers 58G6 and 55A8 exhibited synergetic potency and breadth against several Omicron variants.

### Synergetic neutralizing mechanism of this cocktail.

**Figure 2:**
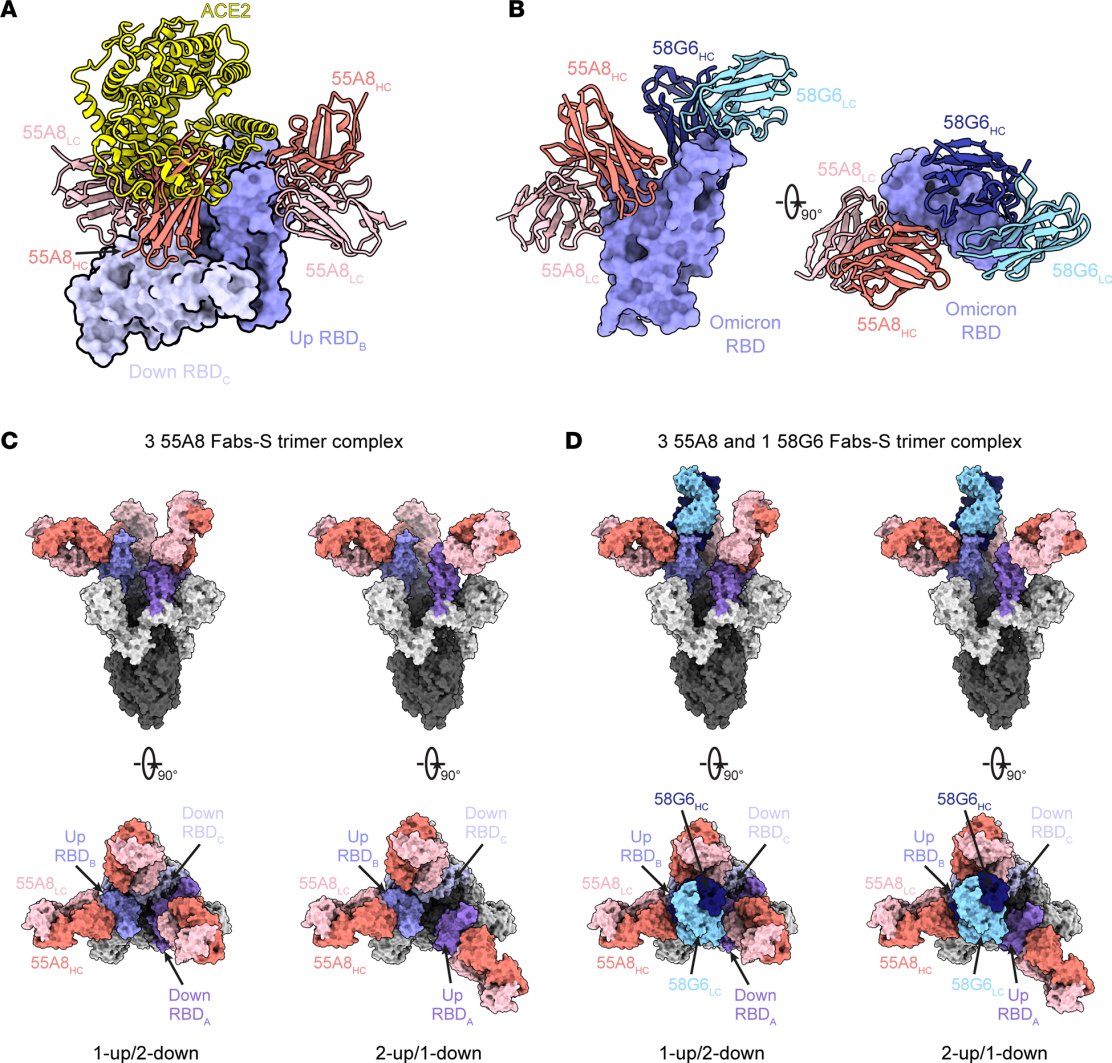
Synergetic neutralizing mechanism of the cocktail of 58G6 and 55A8. (**A**) Superposition of the locally refined Omicron BA.1 RBD-ACE2 (PDB ID: 7WSA) model together with the locally refined Omicron RBD-55A8 Fab model. (**B**) Locally refined model of the 55A8 Fab and 58G6 Fab on the same up Omicron BA.1 RBD. HC, heavy chain; LC, light chain. (**C**) 55A8 Fabs bind to Omicron BA.1 S trimers in 2 states. Two perpendicular views of Omicron BA.1 S-55A8 complexes are shown as the surface. (**D**) 55A8 and 58G6 Fabs simultaneously bind to Omicron BA.1 S trimers in 2 states. Two perpendicular views of Omicron BA.1 S-55A8/58G6 complexes are shown as the surface. 55A8 heavy chain, salmon; 55A8 light chain, pink; 58G6 heavy chain, navy; 58G6 light chain, sky blue; 3 Omicron BA.1 RBDs, different shades of purple.

To investigate the neutralizing mechanism of 55A8, we first studied the single-particle cryo–electron microscopy (cryo-EM) structures of the antigen-binding fragments (Fabs) of 55A8 in complex with the prefusion Omicron BA.1 S trimer (Supplemental Figure 3A). In all observed 55A8 Fab-S complexes, the S trimer adopted a “1-up/2-down” or a “2-up/1-down” conformation (Supplemental Figure 3, A and B). Previous studies have shown that the hinge-like movement of the receptor binding domain (RBD) generates two distinct conformational states of RBD, namely the “up” RBD and the “down” RBD. The “up” RBD represents a conformation that is accessible to the receptor, whereas the “down RBD” corresponds to a conformation that is inaccessible to the receptor (41, 42). Superimposition of the down receptor binding domain (RBD) in the structures of the 55A8 Fab-S and ACE2-S complexes revealed that 55A8 Fab binding with a down RBD could create a steric clash between ACE2 and the adjacent up RBDs, while no overlapping between the 55A8 Fab and ACE2 on the same up RBD were observed (Figure 2A).

Next, the cryo-EM structures of the Omicron BA.1 S trimer in complex with the 55A8 and 58G6 Fabs were determined, and similar conformation of the BA.1 S trimer was observed when treated with the NAb cocktail (Supplemental Figure 3C). A refinement to an overall resolution of 3.3 Å showed that the majority of selected particle images represented a 4-Fab-per-trimer complex, containing three 55A8 Fabs and one 58G6 (Supplemental Figure 3D). Specifically, we found that 55A8 and 58G6 Fab simultaneously bound to a single up RBD, which exhibited no conformational changes compared with the 55A8 Fab-BA.1 S complex (Figure 2B). The addition of 58G6 occupied the ACE2 binding site on the 55A8-bound RBD and further occluded the accessibility of the Omicron BA.1 S protein to ACE2 (Figure 2, C and D). This was further evidenced by the ACE2 competition assay, in which 55A8 showed no competition with ACE2 for binding to the Omicron BA.1 RBD but partially competed with ACE2 for binding to the Omicron BA.1 S protein (Supplemental Figure 4A). Also, we confirmed simultaneous binding of 58G6 and 55A8 to the S proteins of SARS-CoV-2, Omicron BA.1, or Omicron BA.2 in a noncompetitive manner (Supplemental Figure 4B). These findings suggested that the synergetic neutralization might be achieved through complementary steric occlusion of ACE2 by the pair of 55A8 and 58G6.

To determine the binding epitopes of these NAbs on Omicron BA.1 S proteins, we assessed the potential hydrogen bonds of 55A8 and 58G6 complementarity determining regions (CDRs) (Supplemental Figure 5, A–D). Specifically, half of the 6 CDRs (CDRH3, CDRL1, and CDRL3) of the 55A8 Fab were found to directly interact with the S^345–352^ and S^440–450^ regions (Supplemental Figure 6, A and B). Several potential hydrogen bonds, including R346, Y351, K440, S443, K444, V445, and N450, were identified at the interface of the 55A8 Fab and Omicron RBD (Supplemental Figure 6C). The CDRs of 58G6 were shown to form unique interactions with the mutated amino acids N477, K478, and R493 within Omicron BA.1 receptor binding motif (RBM), explaining the sustained neutralizing capability of 58G6 against the Omicron BA.1 variants (Supplemental Figure 6D). The exclusiveness and specificity in interactive residues of 55A8 and 58G6 might serve as the molecular basics for the synergetic effect in neutralizing BA.1.

### Intranasal delivery of the NAb cocktail protects hamsters from Omicron challenge.

**Figure 3:**
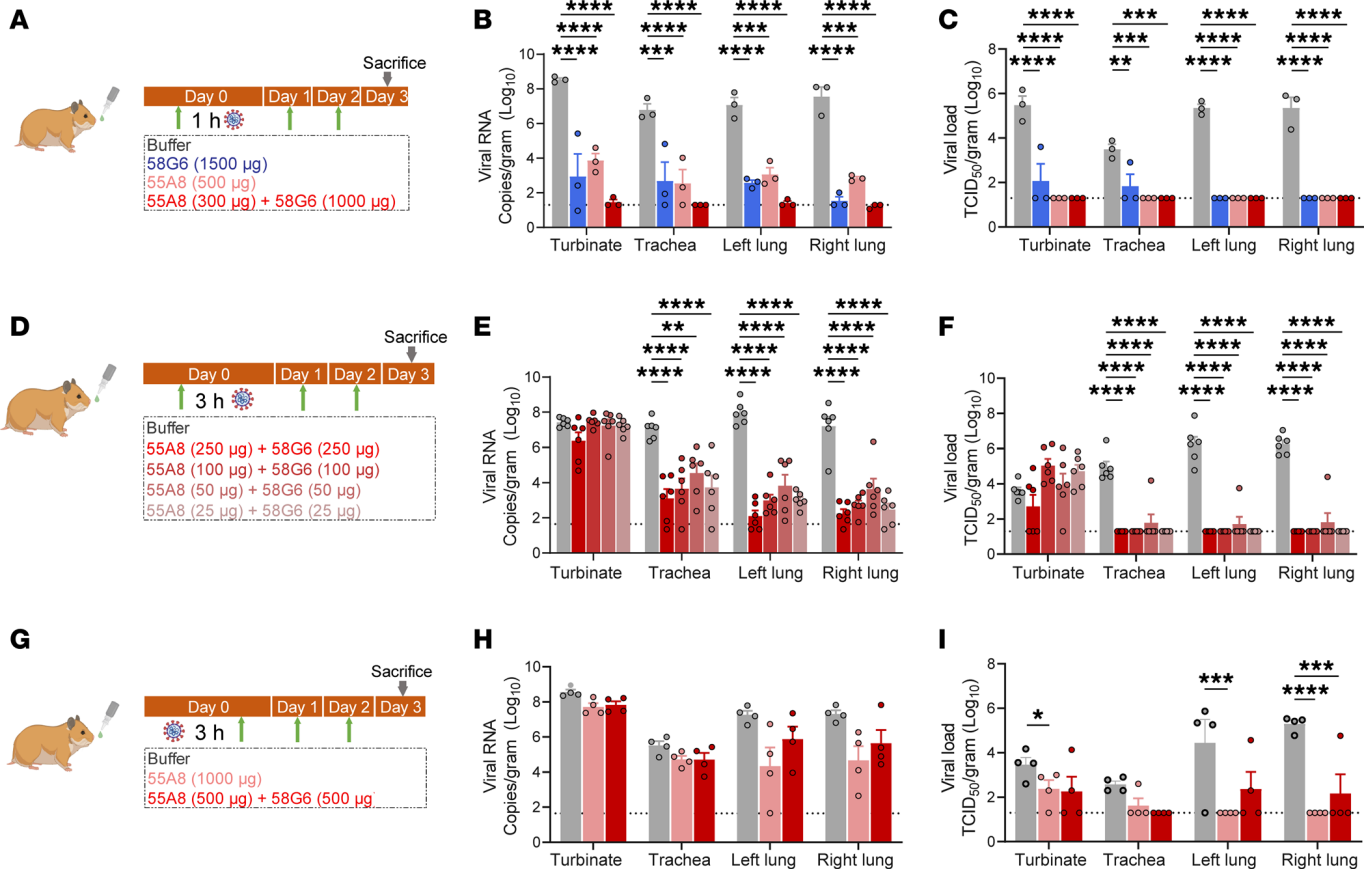
Intranasal delivery of the cocktail of 55A8 and 58G6 protected hamsters from Omicron challenge. Syrian golden hamsters challenged with 1 × 10^4^ PFU of Omicron were treated with 58G6, 55A8, or the 2-antibody cocktail at 1 hour or 3 hours before infection, or 3 hours after infection, with 2 additional treatments at 24 and 48 hours after infection. (**A**, **D**, and **G**) Animal experimental scheme in different sets of experiments and corresponding animal numbers used for each group are shown in **A** (*n* = 3), **D** (*n* = 6), and **G** (*n* = 4). (**B**, **E**, and **H**) The turbinates, trachea, and lungs were harvested on Day 3 after treatment and analyzed by qPCR for viral RNA (log_10_[RNA copies per g]). (**C**, **F**, and **I**) The infectious viruses (PFU/g) in the respiratory tract were measured with a viral plaque assay performed with Vero E6 cells. For all panels, data are shown as individual values with mean ± SEM and analyzed by 1-way ANOVA with Tukey’s multiple-comparison test. *P < 0.05; **P < 0.01; ***P < 0.001; ****P < 0.0001.

The prophylactic or therapeutic protective efficacy of the cocktail containing 55A8 and 58G6 was verified in a hamster model against authentic Omicron BA.1 infection. As demonstrated in Figure 3A, we utilized a dose of 58G6 that exhibited effective neutralizing potency in a previous study (31) and chose the dose of 55A8 as approximately 1:3 in ratio to that of 58G6, due to its comparatively sharp enhance in neutralization activity for pseudotyped or authentic Omicron BA.1. The results of quantitative PCR (qPCR) and plaque assays showed significantly reduced viral RNA copies and infectious virus loads in harvested trachea and lung tissues from animals receiving NAb treatment 1 hour prior to viral challenge and 2 additional dosages on 2 consecutive days after infection (Figure 3, B and C). To this end, both single NAb–treated groups and the combination treatment group demonstrated similar protective effects (Figure 3, B and C).

Next, we explored the sensitivity of the preventative effectiveness of this cocktail by titrating the dosages of the NAb combination in a common 1:1 ratio, with an extended preexposure duration to viral challenge (Figure 3D). Significant decrease in the viral RNA copies was found in the trachea and lung tissues (Figure 3E). The infectious viral load was also reduced substantially, even with a dose of the combination treatment as low as 25 μg of 55A8 and 25 μg of 58G6 (Figure 3, E and F). Encouraged by the observed protective efficacy of the NAb combination, we further analyzed its emergency postinfection treatment potential (Figure 3G). Given the reported difficulty of achieving in vivo therapeutic efficacy (31), we increased the dose of NAbs for this assessment (Figure 3G). Although no apparent rescue of infection in the upper respiratory route was observed, reduced levels of infectious viral load was detected in the lung tissues with the high dose of 1,000 μg of 55A8 alone or a 1:1 ratio of 500 μg 55A8 and 58G6 each (Figure 3, H and I). Collectively, these data from the hamster model show that the combination of 55A8 and 58G6 could confer prophylactic protection even at markedly low doses and a potential applicable value in the emergency treatment through the convenient administration by intranasal delivery.

### Investigator-initiated trial of 55A8 and 58G6 NAb cocktail in healthy volunteers.

**Figure 4:**
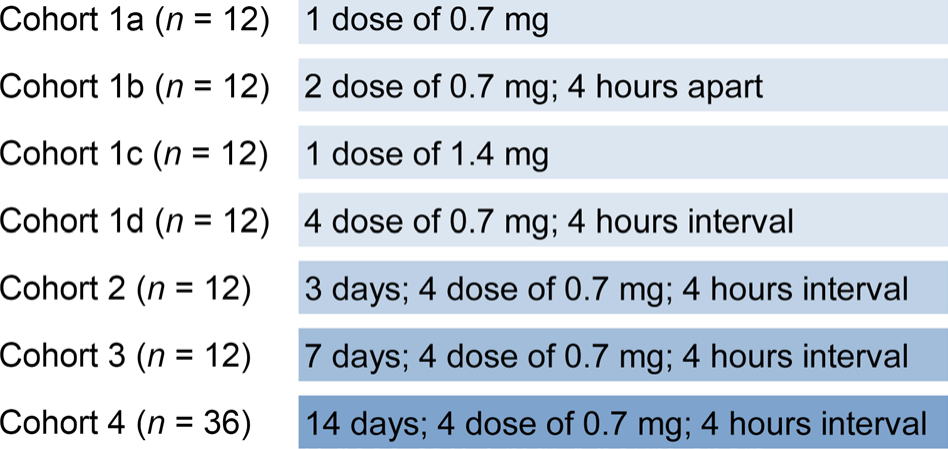
Trial design for the investigator-initiated study of 55A8/58G6 (A8G6) nasal spray neutralization antibody cocktail. A total of 108 healthy volunteers were enrolled in 4 cohorts of study. A8G6: Placebo = 5:1 for each cohort. Cohort 1 has 4 subcohorts, which focused on the study of 1, 2, or 4 doses within 1 day. Cohorts 2, 3 ,and 4 focused on 4-doses-per-day treatment over 3, 7, and 14 days, respectively.

For the safety determination in humans, the cocktail of 55A8 and 58G6 at a 4:1 mass ratio was selected based on the neutralization activity against Omicron BA.5 pseudovirus (Figure 1A). The NAb cocktail was filled in a nasal spray device with a 6 mL sterile vial with aluminum cap crimp over a spray nozzle, which was produced under Good Manufacturing Practice (GMP). A formulation of 5 mg/mL total antibody concentration was utilized for self-administration, termed as the A8G6 antibody cocktail. The composition and concentration of excipients used in the formulation were listed in Supplemental Table 3. Prior to the in-human study, preclinical toxicity of the A8G6 cocktail was analyzed in Rhesus monkeys under Good Laboratory Practice (GLP), and no safety concern was observed (Supplemental Data: Preclinical repeat dosing toxicity studies in nonhuman primates). A randomized and placebo-controlled trial on the intranasal delivery of the NAb cocktail was conducted in 108 healthy volunteers (Figure 4) to assess the safety/tolerability (primary objective) and pharmacokinetics (nasal and serum concentration over time, secondary objective) of A8G6 nasal spray. The baseline demographic information of the participants is listed in Supplemental Table 4, with A8G6 versus placebo as 5:1 in all cohorts. The trial started with Cohort 1, in which different doses ranging from 1 dose to 4 doses were given per day (Figure 4). In cohorts 2, 3, and 4, 4 doses per day were given for 3, 7, and 14 consecutive days, respectively. Overall, A8G6 nasal spray was shown to be well tolerated with minimum treatment-related adverse effects (Supplemental Tables 5 and 6), and the primary objective was met.

**Figure 5:**
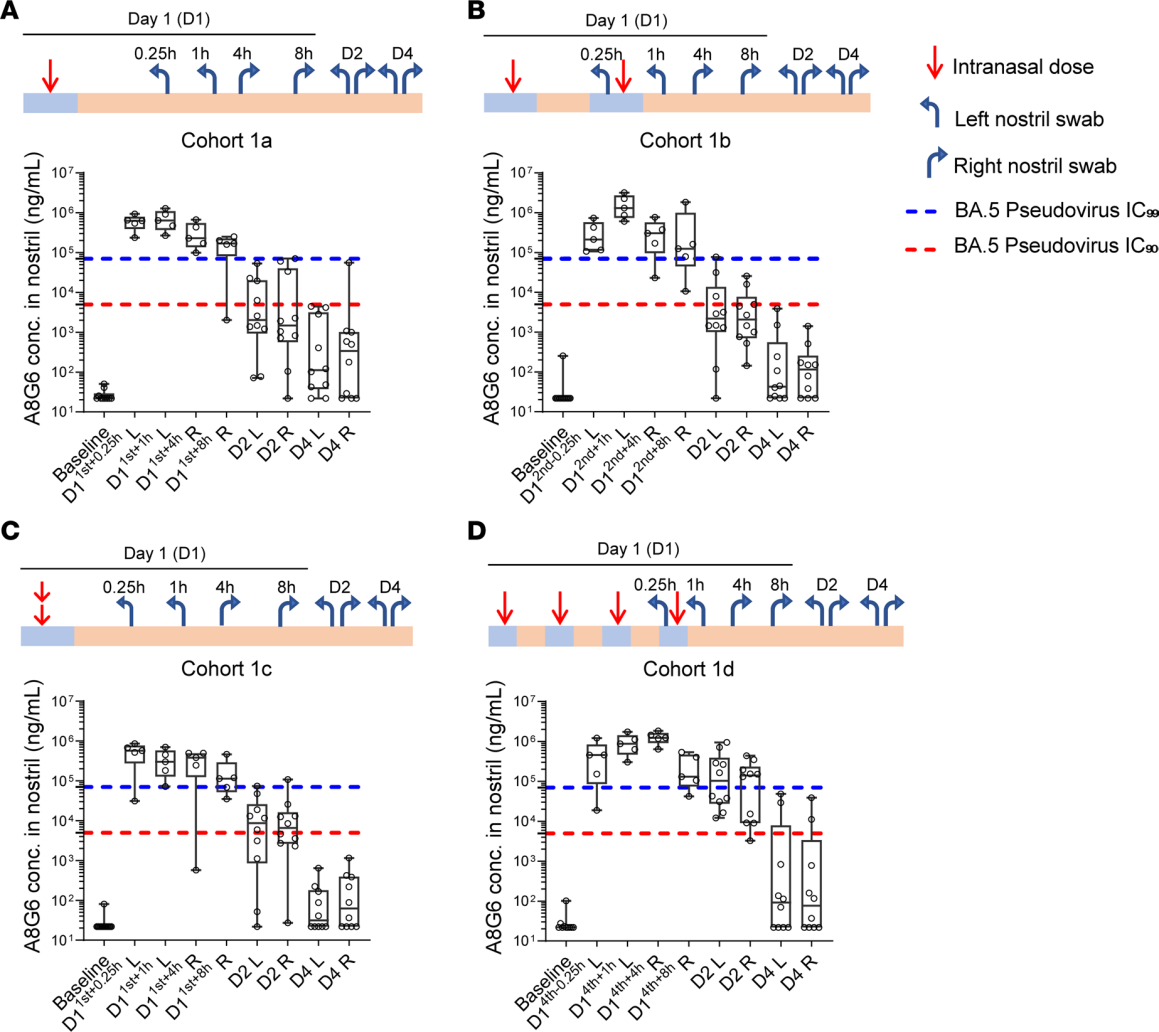
Cohort 1 clinical study of the A8G6 cocktail showed nasal NAbs concentration above the IC_90_ neutralization activity against pseudotyped Omicron BA.5. Operational scheme was listed for each cohort study, showing administration of 1, 2, or 4 doses of A8G6 (each dose containing 560 μg of 55A8 and 140 μg of 58G6 to make a total of 0.7 mg) intranasally (red arrow), sampling time (h, hours; D, day) and from the left or the right nostril (blue arrow). (**A**–**D**) ELISA of washed nasal samples to detect A8G6 nostril concentrations of cohorts 1a–1d. Baseline samples were taken 24 hours prior to the first dosing. All samples were obtained with an indicated time window after the last dose, except for the 0.25-hour time point in **B** and **D**, for which sampling took place 0.25 hours prior to the final dosing. Data are presented in box-and-whiskers plots with maximal and minimal data points (whiskers) and medians (lines). Dashed lines indicate IC_90_ and IC_99_ established earlier from the pseudoviral neutralization assay of BA.5.

**Figure 6:**
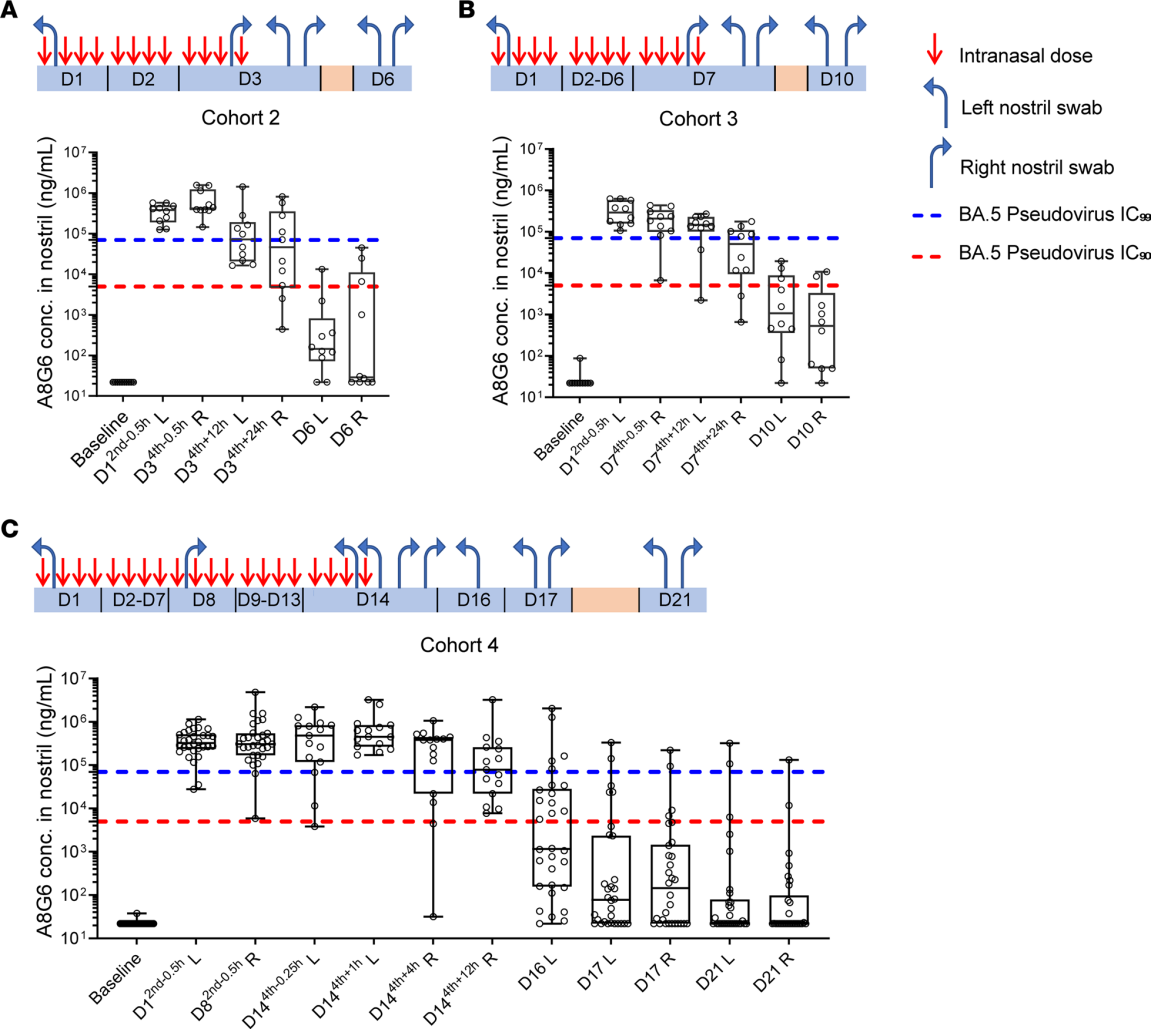
Clinical study of A8G6 cocktail showed nasal NAbs concentration above the IC_90_ neutralization activity on pseudotyped Omicron BA.5 in cohorts 2–4. Operational scheme was listed for cohort 2–4, showing intranasal administration (bold red arrow) of 4 doses of 0.7 mg A8G6 per day over 3 days (*n* = 12), 7 days (*n* = 12), and 14 days (*n* = 36), respectively. In Cohort 2 and Cohort 3, nasal samples were taken 0.5 hours before the second dose on Day 1 and before the last dose on the last day, with indicated side of the nostril (blue arrow). For the postadministration period, sampling was taken 12, 24, and 72 hours after the last dose with indicated side of the nostril. In Cohort 4, nasal samples were taken 0.5 hours before the second dose on Day 1 and Day 8, and 0.25 hours before the last dose. For the postadministration period, samples were taken 1, 4, 12, 48, 72, and 144 hours after the last dose. (**A**–**C**) ELISA of washed nasal samples to detect A8G6 nostril concentrations of cohorts 2–4. Baseline samples were taken 24 hours prior to the first dose. Data are presented in box-and-whiskers plots with maximal and minimal data points (whiskers) and medians (lines). Dashed lines indicate IC_90_ and IC_99_ established earlier from the pseudoviral neutralization assay of BA.5.

For the second objective of the trial, the pharmacokinetics of A8G6 nasal spray was studied to optimize the dosing regimen. The target concentration was defined as nasal concentration above BA.5 pseudovirus neutralization of 90% IC (IC_90_), which was about 5,000 ng/mL (Figure 1A). The results showed that, after a single dose of 0.7 mg A8G6 via nasal administration, nasal concentration of the NAbs remained above pseudotyped BA.5 neutralization IC_90_ for 8 hours in over 90% of participants and dropped below the IC_90_ 24 hours after the single dosing in over 50% of the tested participants (Figure 5A). This suggested the necessity of multiple doses to acquire sufficient nasal concentrations of A8G6 throughout the entire day. In the parallel study in Cohort 1b, 2 doses of nasal spray separated by 4 hours demonstrated similar nasal concentrations 8 hours after the second dose, yet they failed to provide 24-hour A8G6 nasal coverage (Figure 5B). When the second dose of 0.7 mg was delivered immediately after the first dose, the pharmacokinetics of A8G6 nasal concentrations were similar to those observed in Cohort 1a with a single dose (Figure 5, A and C). However, 4 doses of A8G6 administrated 4 hours apart as in Cohort 1d were shown to maintain the NAbs nasal concentration above pseudotyped BA.5 IC_90_ at 24 hours after the first dose (Figure 5D). These data from Cohort 1 suggest that repeat dosing during the day might be effective in providing sustained NAb enrichment at the site of infection. Furthermore, we tested for the nasal concentration of A8G6 after different time courses of administration. With 4-dose-per-day delivery of A8G6 lasting for 3, 7, or 14 consecutive days, we found that the nasal levels of the NAbs did not exceed those detected for the single-day treatment (Figure 6, A–C). The minimum accumulation of A8G6 in nasal cavity over multiple days of dosing suggested a rapid mucosal clearance of these NAbs.

**Figure 7:**
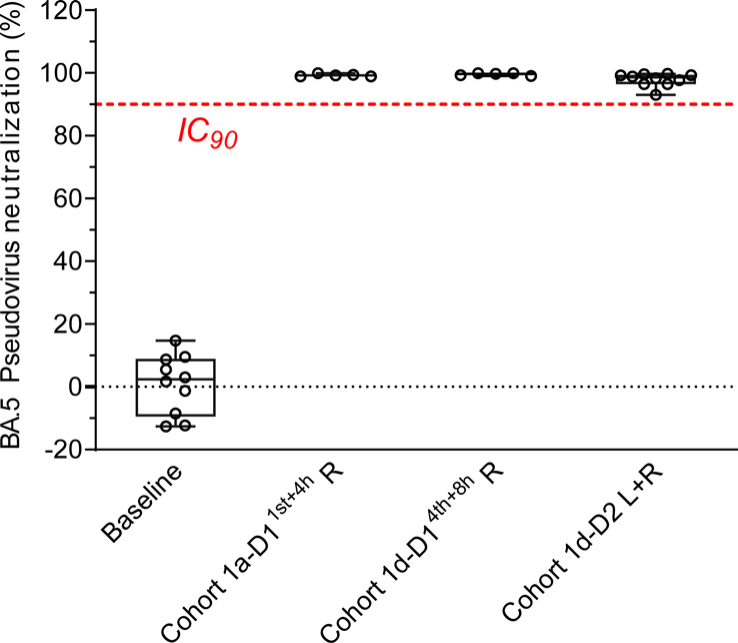
Nasal samples from Cohort 1a and 1d demonstrated over 90% neutralization potency on pseudotyped Omicron BA.5. Nasal swabs samples taken 4 hours after the first dosing in Cohort 1a (*n* = 5), together with samples taken 8 (*n* = 5) and 24 (*n* = 10) hours after the last dosing in Cohort 1d, were subjected to a pseudovirus neutralization assay against BA.5. Baseline samples were taken 24 hours prior to the first dosing in Cohort 1d (*n* = 10). The final neutralization activities in the original nasal samples were calculated by fitting a standard dose response curve. Data are presented in a box-and-whiskers plot with maximal and minimal data points (whiskers) and median (line). Dashed lines indicate IC_90_ of the pseudoviral neutralization assay of BA.5.

In addition, we studied the pharmacodynamics of A8G6 cocktail nasal spray by testing the ex vivo neutralization activity of the nasal mucosal samples against pseudotyped Omicron BA.5. To cover a 24-hour period, we chose samples taken 4 hours after the single dose in Cohort 1a, together with samples taken 8 hours or 24 hours after the fourth dose in Cohort 1d. The results confirm that all samples exhibited potent neutralization activity, as shown by the over 90% inhibition rate against pseudotyped BA.5 (Figure 7). These results indicate that the strategy of repeated nasal administration of A8G6 per day could efficiently provide a protective window of 24 hours.

In order to distinguish and characterize the serum distribution of A8G6, we immunized mice with the Fabs of 58G6 and successfully obtained a pair of anti-58G6 antibodies through a specific binding screening. Consequently, we developed an ELISA for 58G6-targeted detection to analyze the amount of A8G6 cocktail entering systemic blood circulation (Supplemental Figure 7A). The results show that, for participants in cohorts 3 and 4 who received extended days of A8G6 nasal administration, the serum 58G6 concentrations either at 24 hours prior to the first dose or at 1 day after the final dose were below the detection limit of 0.5 ng/mL (Supplemental Figure 7, A–C). This suggests that nasal spray could keep the dispensing of A8G6 at the regional route of infection, without a vast distribution in the systemic blood circulation. To summarize, this human nasal A8G6 cocktail spray provides a good basis for potential clinical efficacy in preventing Omicron BA.5 infections.

## Discussion

Given the identified rapid loss of protection by vaccination against SARS-CoV-2, on-time NAb injection may provide timely prophylactic efficacy, while limited by the high cost and inconvenience in administration (20–22). In this study, we identified 2 synergetic NAbs that could broadly neutralize the emerging Omicron variants. The neutralizing synergy was found to largely rely on a unique complimentary addition of a RBM-targeted NAb 58G6 to a non-RBM NAb 55A8 to completely occlude ACE2 accessibility. Such mutual inhibitions could be suitable for managing the occurrence of escape mutations often seen with long-term application (43–45). The pair of 58G6 and 55A8 might greatly support the current lack of functional single NAb in face of Omicron and its subvariants and may put the cocktail at the center stage for the development of clinically effective prophylactic regiments against the Omicron pandemic (46).

Together with the global attempt to provide affordable and accessible prophylactic drugs for interrupting Omicron transmission in community, we have advanced the A8G6 cocktail to animal and human studies and presented the initial investigation by nasal spraying the NAb cocktail. Since the upper air tract is shown to be favored by Omicron, several studies have evidenced that intranasal pretreatment with various regimen forms, including small molecule inhibitors, antisense oligonucleotides (ASO) targeting SARS-CoV-2 RNA genome, anti-ACE2 mAbs, or miniproteins mimicking hACE2, could effectively reduce respiratory virus replication and prevent SARS-CoV-2 infection (28, 30, 32, 33, 47, 48). We proved that the NAb cocktail we identified could confer prophylactic efficacy even at a markedly low dose of 50 μg against authentic Omicron BA.1 challenge in the hamster model, suggesting an economic outlook for the medical cost with its potential wide-range applications. Notably, the cocktail exhibited mild emergency therapeutical efficacy, although a comparatively higher dose to what was effective for preventative was required for the execution of postinfectious viral neutralization. The limited permeability of NAbs through the nasal mucosa could be the reason for the consequential weakened effectiveness of the cocktail in controlling the intramucosal virus replication. This might also be the reason for no visible synergetic window for the emergency therapy with the cocktail compared with 55A8 applied alone. Contrary to the case of prophylactic application, the addition of 58G6 and the mechanistic proficiency of 55A8 in promoting 58G6-mediated blockage of RBD-ACE2 binding could be discouraged in presence of high viral copies after infection. Nevertheless, the nasal enrichment of these NAbs still exhibited a certain degree of functional neutralization after infection of BA.1, possibly through preventing successful viral assembly in the lung tissues.

Major challenges raised with mucosal drug delivery come from the rapid physical clearance by the mucociliary system (49). Indeed, the results of our trial show that high concentration of NAbs could only be maintained for a period of less than 24 hours in the nasal cavity, but the duration was largely improved by repetitive administrations. This suggests the necessity of excellent biocompatibility with mAb products for this type of application. Both 58G6 and 55A8 were originally patient-derived NAbs, with lower immunogenicity over NAbs from other species or designed proteins. Also, we utilized the IgG subtype of NAbs rather than IgAs with preferential mucosal distribution, due to the fact that IgG exhibited operational maturation in large-scale preparation with stable quality control between batches and could minimize the risks of systemic immunotoxicity (32). These might collectively contribute to the detected biocompatibility of the A8G6 nasal spray, shown by the undetectable presence of NAbs in the serum and a lack of nasal immune response to A8G6 (Supplemental Data: Nasal Swab IgA detecting). These findings are encouraging for the concept of employing repetitive intranasal protective measures in the long-term battle against continuously emerging SARS-CoV-2 mutational variants. Future efforts of improving the mucosal half-life of A8G6, such as modification and polymerization of the NAbs and optimization of the current formula, might enhance the in vivo efficacy of this cocktail, providing wider safety margins and reduced frequency of administration.

There are a few limitations with our study. First, due to the lag in the availability of authentic Omicron sublineage variants, in vitro neutralization experiments were performed against WT SARS-CoV-2 and only 2 mutants, Delta and Omicron BA.1. Continuous examination with updated Omicron sublineages is needed to track the antiviral activity of our cocktail against the full landscape of the Omicron clade. Second, the design of the actual ratio between these 2 NAbs could be further optimized to improve virus neutralization capability of this cocktail. Third, the physiological disparities of respiratory systems between human and rodent models raise questions for the observed preventative efficacy of this NAb cocktail.

In conclusion, we present a potent NAb cocktail against Omicron variants with high prophylactic efficacy at low dosage, which can be self-administrated via nasal spray. The verified tolerability, the associated low cost, and the needle-free convenience make it acceptable for potential large-scale application in the general population. Our product represents promising passive NAb interventions that may effectively aid the current prophylactic vaccines to mitigate SARS-CoV-2 transmission and its probable resurgence.

## Methods

### Sex as a biological variable.

Male and female healthy volunteers were enrolled in this study. Healthy volunteers were enrolled in a convenience sample; thus, there were no prespecified design constraints for the numbers of males or females.

### Cells and viruses.

The SARS-CoV-2 WIV04 strain (IVCAS 6.7512) was originally isolated from a patient with COVID-19 in 2019 (GISAID, accession no. EPI_ISL_402124) (50). The SARS-CoV-2 Delta variant (B.1.617.2; GWHBEBW01000000) was provided by Hongping Wei’s laboratory, Wuhan Institute of Virology, Chinese Academy of Science. The SARS-CoV-2 Omicron virus was isolated from a throat swab of a patient in Hong Kong by the Institute of Laboratory Animal Sciences, Chinese Academy of Medical Sciences (CCPM-B-V-049-2112-18). Viral stocks were prepared by propagation in Vero E6 cells (ATCC CRL-1586) in DMEM supplemented with 10% FBS and 1% penicillin and streptomycin (P/S). All the cells were cultured under 5% CO_2_ in a humidified incubator at 37°C. All live virus experiments were performed at the Wuhan Institute of Virology in a biosafety level 3 (BSL3) containment facility under an approved biosafety use authorization.

### Animals.

Female Syrian hamsters (5–6 weeks of age) were purchased from Wuhan Institute of Biological Products Co., Ltd.

### Protein expression and purification.

The expression and purification procedures for the SARS-CoV-2 Omicron S protein were described previously (39). In brief, the gene encoding stabilized Omicron S ECD HexaPro was constructed and expressed using FreeStyle 293-F cells (Thermo Fisher Scientific, R79007). Protein was purified from filtered cell supernatants using Ni Sepharose resin (GE Healthcare, 17526801) before being subjected to additional purification by gel filtration chromatography using a Superose 6 10/300 increase column (GE Healthcare, 17517201) in 1× PBS (pH 7.4).

All the antibodies were described and prepared as previously described (16, 39). In brief, 2 plasmids containing the light chain and heavy chain of antibodies were transiently cotransfected into Expi293 cells (Thermo Fisher Scientific, A14528) at a 1:1 ratio with ExpiFectamine 293 (Thermo Fisher Scientific, A14525). After 7 days, antibodies were purified from filtered cell supernatants using protein G Sepharose and dialyzed into 1× PBS (pH 7.4).

### ELISA.

To determining the binding ability of NAbs with different S proteins, 384 wells ELISA plates (NUNC Clear Flat-Bottom Immuno Nonsterile 384-Well Plates, 464718) were coated with 10 μL/well of S proteins from SARS-CoV-2 WT and different variants (Sino Biological) at 2 μg/mL in PBS, pH 7.4, 4°C overnight. Serially diluted 55A8 or 58G6 solution was added to each well with 10 μL/well and incubated at 37°C for 1 hour. ALP-conjugated anti–human IgG antibody (Thermo Fisher Scientific, A18808, 1:2,000) was used as the detection antibody at 37°C for 30 minutes. For the quantification of bound IgG, PNPP (Thermo Fisher Scientific, 34045) was added, and the absorbance was measured at 405 nm using MultiSkan GO fluoro-microplate reader (Thermo Fisher Scientific).

To analyze of the concentration of A8G6 in serum or nasal lavage fluid, a pair of antibodies 17E2 and 8B3 that can specifically bind to NAb 58G6 (1 of the 2 components of A8G6 NAbs) were identified from mice immunized Fab of NAb 58G6. In the ELISA assay, 17E2 antibody was used to precoat the microtiter plate, and after blocking, it was made into a solid-phase antibody. After adding calibration standard, blank sample, quality control sample, and test sample for incubation, detection antibody Biotin-8B3 was added; after incubation, SA-HRP biotin was added. When the antibody was used, a complex was formed, and HRP catalyzed the substrate TMB to produce a color reaction to generate a blue product, which turned yellow after adding ELISA stop solution to terminate the reaction, and the depth of the color was positively related to the concentration of 58G6 neutralizing antibody in the sample relevant. Dual wavelengths were used to measure the absorbance value of each well of the microplate; the detection wavelength was 450 nm, the calibration wavelength was 630 nm, and the standard curve was fitted with a 4-parameter equation. The weight factor was fixed (no weight factor), and then the concentration of 58G6 in the sample was calculated.

### BLI.

BLI assays were conducted on Octet R2 Protein Analysis System (Fortebio) as previously described (51). Protein biotinylation was performed using the EZ-link NHS-PEO Solid Phase Biotinylation Kit (Pierce, A35358) and purified using MINI Dialysis Unit (Thermo Fisher Scientific). For measurement of the affinities of 55A8 for SARS-CoV-2 and Delta, Omicron BA.1, Omicron BA.2, and Omicron BA.4/5 variants, streptavidin (SA) biosensors (ForteBio) were immersed with biotinylated mAbs to generate capture mAbs. Then, the sensors were immersed in buffer (0.02% Tween-20, 1 mg/mL BSA in PBS) to the baseline. After association with 2-gold diluted proteins (diluted from 50 nM to 3.125 nM), disassociation was conducted.

For the mAb competition experiments, biotinylated S proteins were loaded at 1 nm onto SA biosensors, and mAb association was performed at 20 μg/mL for 300 seconds. For ACE2 competition experiments, the biotinylated RBD and S were loaded at 1 nm and 3 nm, respectively, at 1 μg/mL onto SA biosensors. The first antibody was allowed to associate for 600 seconds at 20 μg/mL, and the second protein (ACE2 [50 μg/mL] or a mixture of equal amounts of antibodies and ACE2) was allowed to associate for 300 seconds.

### Production of pseudovirus.

These experiments were performed as described previously (38–40, 51). In brief, the codon-optimized gene encoding SARS-CoV-2–S (GenBank QVE75681.1), SARS-CoV-2–S^B.1.1.7^ (GenBank QHD43416), SARS-CoV-2–S^P.1^ (GenBank EPI_ISL_2876136), SARS-CoV-2–S^B.1.351^ (GenBank MZ314998), SARS-CoV-2–S^B.1.617^ (Global Initiative on Sharing All Influenza Data [GISAID] EPI_ISL_2876136), SARS-CoV-2–S^B.1.617.2^ GISAID EPI_ISL_4299998), SARS-CoV-2–C.37 (52), and Omicron and its sublineages (16), and with SARS-CoV-2–S C-terminal 19-aa deletion, was synthesized and cloned into the 2 EcoRI restriction sites of pMD2.G vector by Tsingke Biotechnology.

Lenti-X293T cells were grown to 70% confluency before transfection with VSV-G pseudotyped DG-luciferase, pWPXL, and pSPAX2. These cells were cultured overnight at 37°C with 5% CO_2_. DMEM supplemented with 5% FBS, 100 IU/mL of penicillin, and 100 mg/mL of streptomycin was added to the inoculated cells, which were cultured overnight for 48 hours. The supernatant was harvested, filtered by 0.45 mm filter, and centrifuged at 300*g* for 7 minutes to collect the supernatant. They were then aliquoted and stored at –80°C.

### Inhibition of pseudotyped SARS-CoV-2 infection.

These experiments were performed as described previously with a minor change (38–40, 51). In brief, serially diluted mAbs in a volume of 50 μL were incubated with the same volume of Lenti-X293T cell supernatants containing pseudovirus for 1 hour at 37°C. These pseudovirus/antibody mixtures were added to ACE2-expressing Lenti-X293 T cells (293 T/ACE2 cells). After 8 hours, the supernatants were replaced with fresh culture medium. After 24 hours, the luciferase activities of infected 293T/ACE2 cells were detected with the Bright-Luciferase Reporter Assay System (Beyotime, RG055M). The IC_50_ values of the evaluated mAbs were determined with a Varioskan LUX Microplate Spectrophotometer (Thermo Fisher Scientific) and calculated by 4-parameter logistic regression using GraphPad Prism 8.0.

### Neutralizing activity against authentic SARS-CoV-2.

Neutralization titers of antibodies were measured with a plaque reduction neutralization test (PRNT) using authentic SARS-CoV-2, Delta, and Omicron BA.1 (53). Briefly, Vero E6 cells (2.5 × 10^5^) were seeded in each well of 24-well culture plates. The cells were incubated overnight at 37°C with 5% CO_2_. On the following day, each antibody was serially diluted 5-fold in the culture medium, with the highest concentration being 100 μg/mL. The diluted antibody was incubated with an equal volume including 600 PFU/mL SARS-CoV-2 at 37°C for 1 hour, after which the antibody-virus mixtures were inoculated onto preseeded Vero E6 cell monolayers in 24-well plates. After 1 hour of infection, the inoculum was removed, and 100 μL of overlay medium (DMEM supplemented with 0.8% methylcellulose, 2% FBS, and 1% P/S) was added to each well. After incubating the plates at 37°C for 96 hours, the plates were fixed with 8% paraformaldehyde and stained with 0.5% crystal violet. The plaques in each well were counted and normalized to the non–antibody-treated controls to calculate relative infectivity. The values of mAbs concentration that result in 50% plaque reduction (PRNT_50_) were calculated using GraphPad Prism 9.

### Cryo-EM sample preparation and data collection.

To prepare the complex formed by 55A8 and the SARS-CoV-2 Omicron S protein, purified Omicron S was diluted to a concentration of 2.0 mg/mL in PBS (pH 7.4) and incubated with the 55A8 Fab at a molar ratio of 1:5. The mixture was incubated on ice for 1 hour and then subjected to gel filtration chromatography using a Superose 6 10/300 column (GE Healthcare) in 1× PBS (pH 7.4). The complex sample was concentrated to 0.8 mg/mL, and a 2.5 μL aliquot of the mixture was applied to an H_2_/O_2_ glow-discharged, 300-mesh Quantifoil R1.2/1.3 copper grid (Quantifoil, Micro Tools GmbH). The grid was then blotted for 2.5 seconds with a blot force of –1 at 8°C and 100% humidity and plunge-frozen in liquid ethane using a Vitrobot (Thermo Fisher Scientific).

To prepare the complex formed by 55A8, 58G6, and the SARS-CoV-2 Omicron S protein, purified Omicron S was diluted to a concentration of 0.8 mg/mL in PBS (pH 7.4). A total of 10 μL of purified SARS-CoV-2 S was mixed with 0.6 μL of 5 mg/mL 58G6 Fab fragments in PBS and incubated for 15 minutes on ice. Then, 0.7 μL of 4 mg/mL 55A8 Fab fragments in PBS was applied and incubated for 15 minutes on ice. A 3 μL aliquot of the mixture (added with 0.01% DDM) was applied to an H_2_/O_2_ glow-discharged, 300-mesh Quantifoil R1.2/1.3 grid (Quantifoil, Micro Tools GmbH). The grid was then blotted for 3.0 seconds with a blot force of –1 at 8 °C and 100% humidity and plunge-frozen in liquid ethane using a Vitrobot (Thermo Fisher Scientific).

Cryo-EM data sets were collected on a 300 kV Titan Krios microscope (Thermo Fisher Scientific) equipped with a K3 detector (Gatan). The exposure time was set to 2.4 seconds with a total accumulated dose of 60 electrons per Å^2^, which yielded a final pixel size of 0.82 Å. A total of 1,525 micrographs was collected for the Omicron S-55A8 Fab complex in a single session with a defocus range between 1.2 and 2.2 μm using SerialEM (54), while 3,605 micrographs were collected for the Omicron S-55A8 Fab-58G6 Fab complex under conditions similar to those described previously. The statistics for cryo-EM data collection can be found in Supplemental Tables 1 and 2.

### Cryo-EM data processing.

All dose-fractioned images were motion-corrected and dose-weighted with MotionCorr2 software (55), and their contrast transfer functions (CTFs) were estimated by cryoSPARC patch CTF estimation (56). For the data set for the Omicron S-55A8 Fab complex, a total of 847,678 particles was autopicked, and after 2D classification, 262,990 particles were selected for ab-initio reconstruction in 6 classes. These 6 classes were used as 3D volume templates for heterogeneous refinement with all selected particles, with 181,072 particles converged into the Omicron S triple-bound 55A8 class. Next, this particle set was used to perform ab-initio reconstruction again in 4 classes. After heterogeneous refinement with all selected particles, 82,383 particles converged into “1-up/2-down” (41, 42) conformation, and 94,894 particles converged into “2-up/1-down” conformation. Next, these 2 particle sets were used to perform nonuniform refinement, yielding a resolution of 3.4 Å for both triple-bound Omicron S-55A8 Fab complexes.

For the data set for the Omicron S-55A8/58G6 Fab complex, a total of 914,631 particles was autopicked, and after 2D classification, 608,282 particles were selected for several rounds of ab-initio reconstruction in 6 classes. These 6 classes were used as 3D volume templates for heterogeneous refinement with all selected particles. Finally, 216,663 particles converged into “1-up/2-down” conformation, and 204,153 particles converged into “2-up/1-down” conformation. Next, these 2 particle sets were used to perform nonuniform refinement, yielding a resolution of 3.3 Å for both triple-bound Omicron S-55A8 Fab complexes.

To improve the local resolution at the binding interface, we subsequently added local refinement processing. A local reconstruction focusing on 2 adjacent up and down RBDs with one 55A8 Fab bound to each RBD was carried out. Furthermore, the density map for the binding interface could be further improved by local averaging of the RBD-55A8 Fab equivalent copies, ultimately yielding a 3.3 Å map of the region corresponding to the RBD-55A8 Fab interface. Similarly, we improved the local resolution between the 55A8 and 58G6 variable domains and the RBD up to 3.3 Å.

Local resolution estimation, filtering, and sharpening were also carried out using cryoSPARC. The full cryo-EM data processing workflow is described in Supplemental Figure 3, and the model refinement statistics can be found in Supplemental Tables 1 and 2.

### Model building and refinement.

To build the structures of the Omicron S-55A8 Fab complex, the structure of the Omicron S-510A5 complex model (Protein Data Bank [PDB] ID: 7WS5) (51) was placed and rigid-body fitted into cryo-EM electron density maps using UCSF Chimera (57). The 55A8 Fab model was first predicted using Phyre2 (58) and then manually built in Coot 0.9 (59), with the guidance of the cryo-EM electron density maps, and overall real-space refinements were performed using Phenix 1.19 (60).

To build the structures of the Omicron S-55A8/58G6 Fab complex, the previously described structure of the Omicron S-55A8 complex model was placed and rigid-body fitted into cryo-EM electron density maps using UCSF Chimera. The 58G6 Fab model (39) was manually built in Coot 0.9 with the guidance of the cryo-EM electron density maps, and overall real-space refinements were performed using Phenix 1.19.

### Animal protection experiments.

To assess whether the 58G6 and 55A8 antibody cocktail induced synergetic effector function responses in vivo, a hamster viral challenge study was performed. Anesthesia was induced via inhalation with 5% isoflurane in 100% oxygen, and hamsters were infected with 1 × 104 PFU (100 μL/hamster, 50 μL/nostril) of Omicron BA.1. Hamsters were infected with 1 × 10^4^ PFU (100 μL/hamster, 50 μL/nostril) of Omicron BA.1. Antibodies 58G6 and 55A8 (in house) were dissolved in a buffer containing 20 mM PB, 4% (W/V) trehalose, 0.01% (W/V) PS80, 0.1% (W/V) HPMC, and 1.7% (W/V) glycerin, pH 6.0. In Figure 3A, hamsters were treated with 58G6 (1500 μg, 15 mg/mL, 100 μL/hamster, 50 μL/nostril/dose), 55A8 (500 μg, 5 mg/mL, 100 μL/hamster, 50 μL/nostril/dose), or 58G6 + 55A8 mixture (1000 μg of 58G6 at 10 mg/mL; 300 μg of 55A8 at 3 mg/mL; 100 μL mixture/hamster, 50 μL mixture/nostril/dose) (*n* = 3 for each group) at 1 hour before infection and at 24 and 48 hours after infection. On Day 3 after infection, the animals were sacrificed, and the turbinate, trachea, and lung were harvested.

To determine the antibody cocktail dose that could provide protection, we performed additional animal experiments (Figure 3D). A range of doses (500–50 μg; 1:1 ratio of 58G6/55A8; 100 μL/hamster, 50 μL/nostril/dose) of the 2-antibody cocktail were inoculated into hamsters intranasally (*n* = 6 for each group). Three hours later, the golden hamsters were anesthetized with isoflurane and intranasally inoculated with 1 × 10^4^ PFU (100 μL/hamster, 50 μL/nostril) of Omicron BA.1. Then, 2 doses of the antibody cocktail were administered at 24 hours and 48 hours. To investigate the potential postinfection treatment potency of 55A8 and the cocktail (Figure 3G), at 3 hours after infection, animals were given an intranasal dose of 1,000 μg of 55A8 (1,000 μg of 58G6 at 10 mg/mL, 100 μL/hamster, 50 μL/nostril/dose) or the 2-antibody cocktail (500 μg of 58G6 at 5mg/mL, 500 μg of 55A8 at 5mg/mL, 100 μL mixture/hamster, 50 μL/nostril/dose; *n* = 4 for each group). Then, 2 additional doses were administered at 24 hours and 48 hours.

### qPCR assay.

To determine the RNA copy numbers in different tissues, SARS-CoV-2 genomic mRNA was assessed by qPCR as previously described (61) using the following primer and probe sequences: ORF1ab forward, 5′-CCCTGTGGGTTTTACACTTAA-3′, and ORF1ab reverse, 5′-ACGATTGTGCATCAGCTGA-3′.

### Plaque assay for SARS-CoV-2.

The viral titers in different tissues were determined with a plaque assay performed as previously described with slight modification (62). Briefly, virus samples were 10-fold serially diluted in culture medium (first dilution, 1:5) and inoculated onto Vero E6 cells seeded overnight at 1.5 × 10^5^/well in 24-well plates. After a 1-hour incubation at 37°C, the inoculum was replaced with DMEM containing 2.5% FBS and 0.9% carboxymethyl-cellulose. The plates were fixed with 8% paraformaldehyde and stained with 0.5% crystal violet 4 days later.

### Investigator-initiated trial of 55A8/58G6 (A8G6) antibody cocktail in healthy volunteers.

The trial of A8G6 nasal spray was an investigator-initiated, double-blinded, randomized, and placebo-controlled trial conducted at the Second Affiliated Hospital of Chongqing Medical University. Eligible participants were randomly assigned to receive either A8G6 nasal spray or placebo nasal spray. An independent data safety monitoring committee performed trial oversight and made recommendations after review of safety reports between cohorts. Full details of the trial design, conduct, oversight and sample analysis, and statistical analysis were provided in the protocol, which is available in the Supplemental Data (A8G6 IIT protocol).

In total, 108 healthy volunteers were enrolled in the trial. Baseline demographic information is provided in Supplemental Table 4. Trial participants were randomly assigned at a 10:2 ratio to receive A8G6 nasal spray or placebo nasal spray. Each dose contained 700 μg of antibody cocktail (560 μg of 55A8 plus 140 μg of 58G6) delivered intranasally at 70 μL to left nostril and 70 μL to right nostril. Trial design is listed in Figure 4. After screening, eligible participants were randomly assigned to Cohort 1, Cohort 2, Cohort 3, and Cohort 4, and blood and nasal swabs were collected for drug concentration testing at the corresponding time points from 1 day prior to the first dose to 3 ± 1 days after the last dose (7 ± 2 days for Cohort 4); safety testing was performed on Day 2 and 3 ± 1 days after the last dose for Cohort 1, on Day 4 and 3 ± 1 days after the last dose for Cohort 2, on Day 8 and 3 ± 1 days after the last dose for Cohort 3, and on Day 17 and 7 ± 2 days after the last dose for Cohort 4. Cohort 1 underwent safety testing on Day 2 and 3 ± 1 days after the last dose, Cohort 2 on Day 4 and 3 ± 1 days after the last dose, Cohort 3 on Day 8 and 3 ± 1 days after the last dose, and Cohort 4 on Day 17 and 7 ± 2 days after the last dose.

The institutions participating in clinical trials and each inspection method have corresponding quality control standards to ensure the quality control of clinical trials and the implementation of quality assurance system. To ensure the reliability and integrity of the trial data, the supervisors appointed by the sponsor regularly conduct systematic monitoring of the clinical institution to determine whether the execution of the trial is consistent with the trial protocol and whether the reported data are consistent with the original information.

Data management, statistical analysts, and researchers worked together to conduct a data review, determine data set delineation, and discuss and finalize the Statistical Analysis Plan (SAP). The database was locked, and then the statistical analysis was calculated. Statistical analysis was completed using SAS (version 9.3 or above) software.

The primary objective is the safety/tolerability of A8G6 nasal spray. The number of cases and incidence of adverse events, adverse reactions, serious adverse events, and adverse events leading to withdrawal from the trial were calculated; the number of cases and incidence were counted according to severity; and the number of cases and incidence were counted according to the relationship with the study drug. Detailed listings of adverse events, adverse reactions, and serious adverse events — including drug number, name of adverse event, onset date, end date, duration, severity, relationship to study drug, action taken, and outcome — were recorded. Classification was done by System Organ Class/Preferred Term (SOC/PT) according to MedDRA terminology codes and included listing the classification details and counting the number of cases and incidence of adverse events according to the system.

The secondary objective is the pharmacokinetics of A8G6 nasal spray, which is the concentration of A8G6 over time in both nasal cavity and blood serum. Nasal swabs (containing approximately 50 μL of nasal surface mucus) were washed in 500 μL PBS buffer, resulting in an 11-fold dilution. The concentration of A8G6 was determined using as a specific ELISA as mentioned above. The selected washed solutions were further diluted 10-fold, 20-fold, or 40-fold in cell culture media to prepare them for in vitro neutralization assays. The neutralization activities of the diluted samples were measured using the Omicron BA.5 pseudovirus neutralization assay mentioned above. The percentage of neutralization activity of nasal swabs was estimated by fitting the standard curve of A8G6 and was averaged cross 3 dilutions (10-fold, 20-fold, and 40-fold).

### Statistics.

Statistical analyses of the animal studies were performed using GraphPad Prism software v.9.2.0. Comparisons among multiple groups were performed using 1-way ANOVA followed by Tukey’s multiple-comparison post hoc test. *P* < 0.05 was considered significant.

### Study approval.

All the animal studies were reviewed and approved by the IACUC of the Institute of Wuhan Institute of Virology, Chinese Academy of Sciences, and performed in an ABSL-3 facility (WIVA45202104). The human trial was conducted in accordance with the Second Affiliated Hospital of Chongqing Medical University (Chongqing, China) Institutional Ethics Review Board (AY-62-8001). The ethical approval of this trial complies with the requirements of the Good Clinical Practice, the Declaration of Helsinki of the World Medical Association, International Ethical Guidelines on Biomedical Research Involving Human Subjects of the Council for International Organizations of Medical Sciences, and relevant domestic laws and regulations. Written informed consent was obtained for all study participant.

### Data availability.

The coordinates and cryo-EM map files for the 55A8-BA.1 S complexes and 55A8/58G6-BA.1 S complexes have been deposited in the PDB under accession nos. 7WWI, 7WWJ, 7WWK, 7XJ6, 7XJ8, and 7XJ9. The study protocol of the investigator-initiated trial and other PK/PD data are provided in the Supplemental Data (A8G6 IIT protocol). Values for all data points in graphs are reported in the Supporting Data Values file. All other data are available from the corresponding author upon reasonable request.

## Author contributions

AH, SC, AJ, HY, GC, and CY conceived and designed the project. For biological function analysis of the NAbs, FL, T Li, MS, XH, YW, and CH screened and cloned the antibodies and expressed and purified the antibodies. FL, T Li, and WW were responsible for BLI assays for the binding ability, affinity, and the competition experiment of NAbs. FL, T Li, SS, KW, NT, MD, and SL prepared various pseudovirus and conducted the pseudovirus neutralization assays. For the efficacy test of the NAbs in vitro and in vivo, X Zhang, HZ, JZ, SC, YW, and RG performed authentic SARS-CoV-2 neutralization assays and animal experiments. For structure analysis, HG and YL cloned, expressed, and purified Omicron BA.1 S proteins. HG, YG, and YL collected, processed the cryo-EM data and built and refined the structure model. XJ, HY, and TJ analyzed and discussed the cryo-EM data. DZ, AH, MD, SL, CL, T Lu, BL, YT, CY, and GC designed and supported the investigator-initiated trial. AH, SC, AJ, XJ, RG, X Zhao, FL, YT, and GC wrote the manuscript. All authors revised and reviewed the manuscript. The authorship order among co–first authors was assigned based on workload and manuscript preparation.

## Supplementary Material

Supplemental data

ICMJE disclosure forms

Supporting data values

## Version 1 04/08/2024

Electronic publication

## References

[1] Mlcochova P (2021). SARS-CoV-2 B.1.617.2 Delta variant replication and immune evasion. Nature.

[2] Wang P (2021). Antibody resistance of SARS-CoV-2 variants B.1.351 and B.1.1.7. Nature.

[3] Wang P (2021). Increased resistance of SARS-CoV-2 variant P.1 to antibody neutralization. Cell Host Microbe.

[4] Meng B (2022). Altered TMPRSS2 usage by SARS-CoV-2 Omicron impacts infectivity and fusogenicity. Nature.

[5] Zhao H (2022). SARS-CoV-2 Omicron variant shows less efficient replication and fusion activity when compared with Delta variant in TMPRSS2-expressed cells. Emerg Microbes Infect.

[6] Hui KPY (2022). SARS-CoV-2 Omicron variant replication in human bronchus and lung ex vivo. Nature.

[7] Carvalho T (2020). Silent spread. Nat Med.

[9] Garrett N (2022). High asymptomatic carriage with the Omicron variant in South Africa. Clin Infect Dis.

[10] Andrews N (2022). Duration of protection against mild and severe disease by Covid-19 vaccines. N Engl J Med.

[11] Andrews N (2022). Effectiveness of COVID-19 booster vaccines against COVID-19-related symptoms, hospitalization and death in England. Nat Med.

[12] Burki TK (2022). Omicron variant and booster COVID-19 vaccines. Lancet Respir Med.

[13] Kuhlmann C (2022). Breakthrough infections with SARS-CoV-2 omicron despite mRNA vaccine booster dose. Lancet.

[14] Costa Clemens SA (2022). Heterologous versus homologous COVID-19 booster vaccination in previous recipients of two doses of CoronaVac COVID-19 vaccine in Brazil (RHH-001): a phase 4, non-inferiority, single blind, randomised study. Lancet.

[15] Reynolds CJ (2022). Immune boosting by B.1.1.529 (Omicron) depends on previous SARS-CoV-2 exposure. Science.

[16] Cao Y (2022). BA.2.12.1, BA.4 and BA.5 escape antibodies elicited by Omicron infection. Nature.

[17] Mahase E (2021). Covid-19: Pfizer’s paxlovid is 89% effective in patients at risk of serious illness, company reports. BMJ.

[18] Burki T (2022). The future of Paxlovid for COVID-19. Lancet Respir Med.

[19] Phizackerley D (2022). Three more points about Paxlovid for covid-19. BMJ.

[20] The Lancet Infectious Diseases (2021). Unmet need for COVID-19 therapies in community settings. Lancet Infect Dis.

[21] Dolgin E (2021). The race for antiviral drugs to beat COVID - and the next pandemic. Nature.

[22] Rubin EJ (2021). Audio interview: a potential new agent to treat Covid-19. N Engl J Med.

[23] Levin MJ (2022). Intramuscular AZD7442 (tixagevimab-cilgavimab) for prevention of Covid-19. N Engl J Med.

[24] Loo YM (2022). The SARS-CoV-2 monoclonal antibody combination, AZD7442, is protective in nonhuman primates and has an extended half-life in humans. Sci Transl Med.

[25] Stuver R (2022). Activity of AZD7442 (tixagevimab-cilgavimab) against Omicron SARS-CoV-2 in patients with hematologic malignancies. Cancer Cell.

[26] Rubin R (2022). Questions remain about who will get monoclonal antibodies for COVID-19 preexposure prophylaxis. JAMA.

[27] Rubin R (2021). Monoclonal antibodies for COVID-19 preexposure prophylaxis can’t come fast enough for some people. JAMA.

[28] Hunt AC (2022). Multivalent designed proteins neutralize SARS-CoV-2 variants of concern and confer protection against infection in mice. Sci Transl Med.

[29] Fan W (2022). Nasal delivery of thermostable and broadly neutralizing antibodies protects mice against SARS-CoV-2 infection. Signal Transduct Target Ther.

[30] Ou J (2022). ACE2-targeting antibody suppresses SARS-CoV-2 Omicron and Delta variants. Signal Transduct Target Ther.

[31] Zhang X (2022). A potent neutralizing antibody provides protection against SARS-CoV-2 Omicron and Delta variants via nasal delivery. Signal Transduct Target Ther.

[32] Ku Z (2021). Nasal delivery of an IgM offers broad protection from SARS-CoV-2 variants. Nature.

[33] Wu X (2021). A potent bispecific nanobody protects hACE2 mice against SARS-CoV-2 infection via intranasal administration. Cell Rep.

[34] Parray HA (2021). Inhalation monoclonal antibody therapy: a new way to treat and manage respiratory infections. Appl Microbiol Biotechnol.

[35] Bequignon E (2019). FcRn-dependent transcytosis of monoclonal antibody in human nasal epithelial cells in vitro: a prerequisite for a new delivery route for therapy?. Int J Mol Sci.

[36] Al Ojaimi Y (2022). Therapeutic antibodies - natural and pathological barriers and strategies to overcome them. Pharmacol Ther.

[37] Sockolosky JT (2012). Engineering neonatal Fc receptor-mediated recycling and transcytosis in recombinant proteins by short terminal peptide extensions. Proc Natl Acad Sci U S A.

[38] Han X (2021). A rapid and efficient screening system for neutralizing antibodies and its application for SARS-CoV-2. Front Immunol.

[39] Li T (2021). Potent SARS-CoV-2 neutralizing antibodies with protective efficacy against newly emerged mutational variants. Nat Commun.

[40] Gao F (2021). A highly conserved peptide vaccine candidate activates both humoral and cellular immunity against SARS-CoV-2 variant strains. Front Immunol.

[41] Wrapp D (2020). Cryo-EM structure of the 2019-nCoV spike in the prefusion conformation. Science.

[43] Rockett R (2022). Resistance mutations in SARS-CoV-2 Delta variant after sotrovimab use. N Engl J Med.

[44] Ku Z (2021). Molecular determinants and mechanism for antibody cocktail preventing SARS-CoV-2 escape. Nat Commun.

[45] Sonnleitner ST (2022). Cumulative SARS-CoV-2 mutations and corresponding changes in immunity in an immunocompromised patient indicate viral evolution within the host. Nat Commun.

[46] Li D (2022). SARS-CoV-2 neutralizing antibodies for COVID-19 prevention and treatment. Annu Rev Med.

[47] Shapira T (2022). A TMPRSS2 inhibitor acts as a pan-SARS-CoV-2 prophylactic and therapeutic. Nature.

[48] Zhu C (2022). An intranasal ASO therapeutic targeting SARS-CoV-2. Nat Commun.

[49] Ibrahim M (2015). Inhalation drug delivery devices: technology update. Med Devices (auckl).

[50] Zhou P (2020). A pneumonia outbreak associated with a new coronavirus of probable bat origin. Nature.

[51] Guo H (2022). Structures of Omicron spike complexes and implications for neutralizing antibody development. Cell Rep.

[52] Kimura I (2022). The SARS-CoV-2 Lambda variant exhibits enhanced infectivity and immune resistance. Cell Rep.

[53] Zhao S (2021). Identification of potent human neutralizing antibodies against SARS-CoV-2 implications for development of therapeutics and prophylactics. Nat Commun.

[54] Mastronarde DN (2005). Automated electron microscope tomography using robust prediction of specimen movements. J Struct Biol.

[55] Zheng SQ (2017). MotionCor2: anisotropic correction of beam-induced motion for improved cryo-electron microscopy. Nat Methods.

[56] Punjani A (2017). cryoSPARC: algorithms for rapid unsupervised cryo-EM structure determination. Nat Methods.

[57] Pettersen EF (2004). UCSF Chimera--a visualization system for exploratory research and analysis. J Comput Chem.

[58] Kelley LA (2015). The Phyre2 web portal for protein modeling, prediction and analysis. Nat Protoc.

[59] Emsley P (2010). Features and development of Coot. Acta Crystallogr D Biol Crystallogr.

[60] Liebschner D (2019). Macromolecular structure determination using X-rays, neutrons and electrons: recent developments in Phenix. Acta Crystallogr D Struct Biol.

[61] Feng L (2020). An adenovirus-vectored COVID-19 vaccine confers protection from SARS-COV-2 challenge in rhesus macaques. Nat Commun.

[62] Zhang YN (2020). A mouse model for SARS-CoV-2 infection by exogenous delivery of hACE2 using alphavirus replicon particles. Cell Res.

