# Supplementary material for: Prophylactic efficacy of an intranasal spray with 2 synergetic antibodies neutralizing Omicron
Source: JCI Insight. 2024 Apr 8;9(7):e171034. doi: 10.1172/jci.insight.171034 (PMC11128199; doi:10.1172/jci.insight.171034)
Supplement: Supplemental data [file jciinsight-9-171034-s236.pdf]

Supplemental Figures

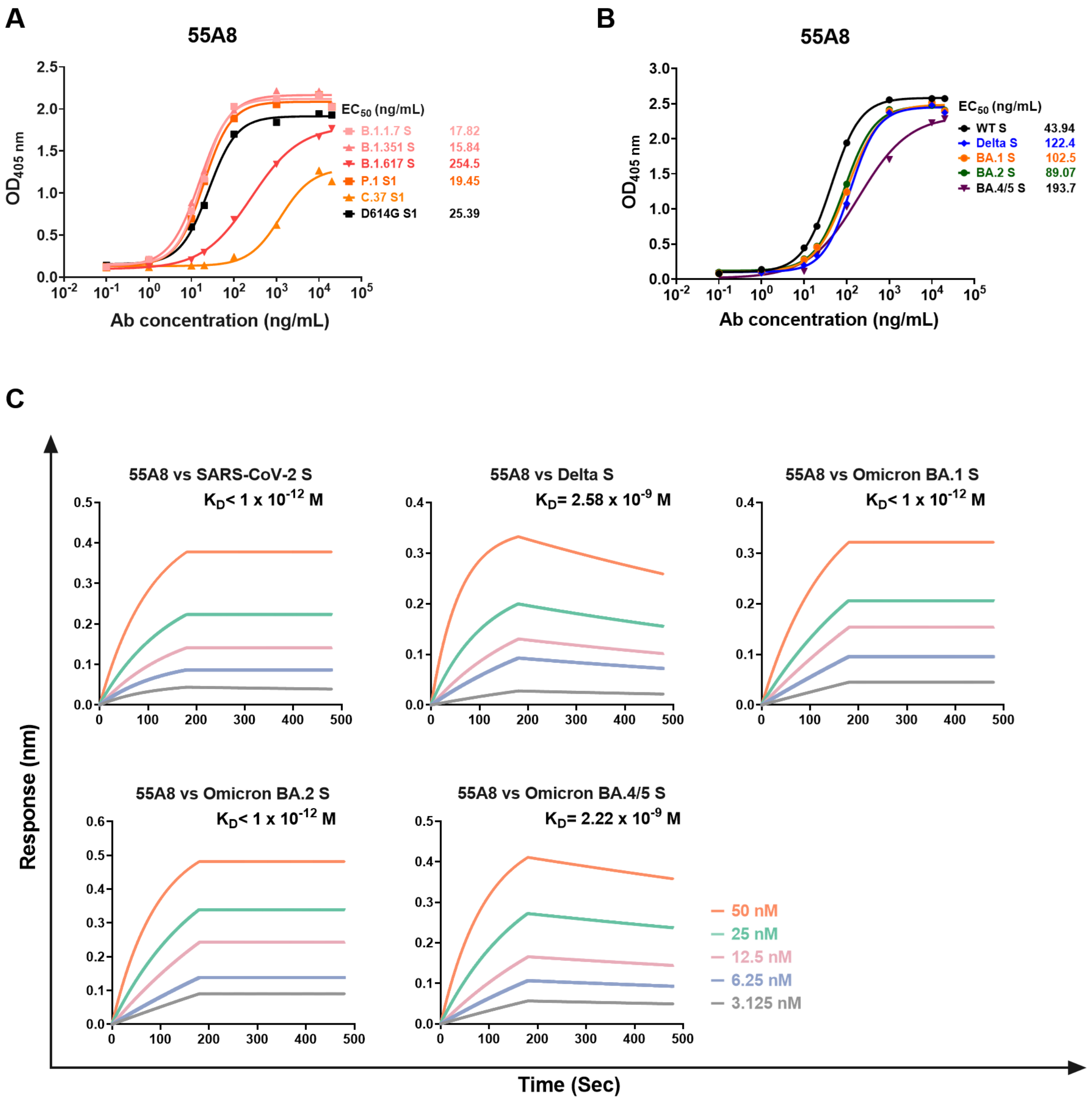

Supplemental Figure 1. The binding capabilities of 55A8 against the Spike proteins of several SARS-CoV-2 variants. (**A-B**), The binding capabilities of 55A8 against the S or S1 proteins of several SARS-CoV-2 variants including WT-type, D614G, Alpha (B.1.1.7), Beta (B.1.351), Kappa (B.1.617), Gamma (P.1), Lambda (C.37), Delta (B.1.617.2), Omicron BA.1 (B.1.1.529.1), BA.2 (B.1.1.529.2) and BA.4/5 (BA.1.1.529.4 or BA.1.1.529.5) were measured by ELISA. Data are representative of one independent experiment out of two. (**C**) The affinities of 55A8 with the S proteins of SARS-CoV-2 and Delta, Omicron BA.1, BA.2 and BA.4/5 variants were measured by BLI.

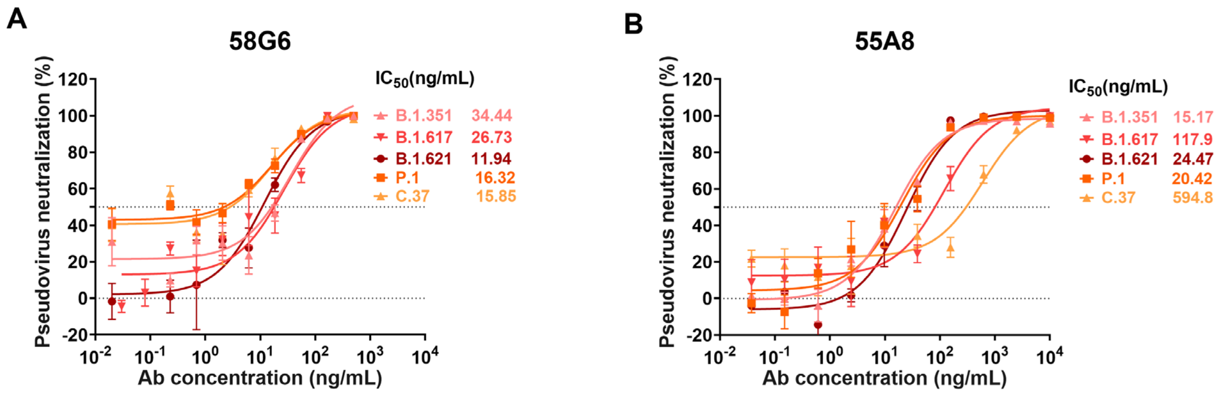

Supplemental Figure 2. The neutralizing potencies of 58G6 and 55A8 against several SARS-CoV-2 variant pseudoviruses. (**A-B**) The neutralizing potencies of 55A8 and 58G6 against the variants B.1.1.7, B.1.351, B.1.617, B.1.621, B.1.617.2, P.1 and C.37 were measured with a pseudovirus neutralization assay. The dashed line indicates a 0% or 50% reduction in viral neutralization. Data for each NAb were obtained from a representative neutralization experiment of three replicates, presented as mean values ± SEM.

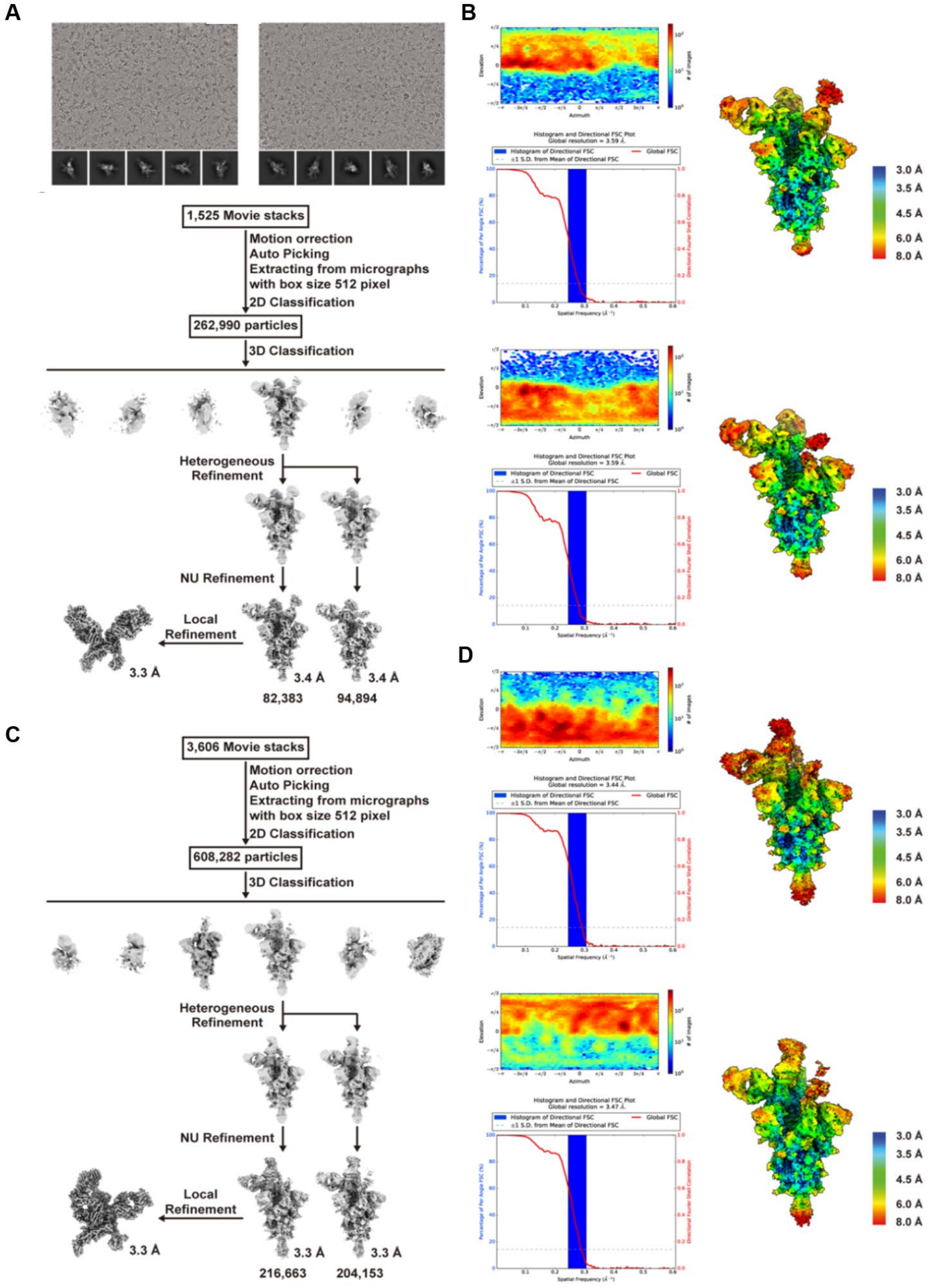
A
1,525 Movie stacks
Motion orrection
Auto Picking
Extracting from micrographs
with box size 512 pixel
2D Classification
262,990 particles
3D Classification
Heterogeneous
Refinement
NU Refinement
Local
Refinement
3.3 Å
3.4 Å
3.4 Å
82,383
94,894
B
Histogram and Directional FSC Plot
Global resolution = 3.59 Å
Histogram of Directional FSC
Global FSC
±1 S.D. from Mean of Directional FSC
Azimuth
Elevation
Spatial Frequency (Å⁻¹)
3.0 Å
3.5 Å
4.5 Å
6.0 Å
8.0 Å
Histogram and Directional FSC Plot
Global resolution = 3.59 Å
3.0 Å
3.5 Å
4.5 Å
6.0 Å
8.0 Å
C
3,606 Movie stacks
Motion orrection
Auto Picking
Extracting from micrographs
with box size 512 pixel
2D Classification
608,282 particles
3D Classification
Heterogeneous
Refinement
NU Refinement
Local
Refinement
3.3 Å
3.3 Å
3.3 Å
216,663
204,153
D
Histogram and Directional FSC Plot
Global resolution = 3.44 Å
3.0 Å
3.5 Å
4.5 Å
6.0 Å
8.0 Å
Histogram and Directional FSC Plot
Global resolution = 3.47 Å
3.0 Å
3.5 Å
4.5 Å
6.0 Å
8.0 Å

Supplemental Figure 3. Cryo-EM data processing workflow for Omicron S in complex with the 55A8 Fab or 55A8 Fab-58G6 Fab. Representative cryo-EM micrographs, 2D classes and workflow for the Omicron S-55A8 Fab (**A**) and Omicron S-55A8/58G6 Fab (**C**) 3D reconstructions**.** (**B**), (**D**) Viewing direction distribution plot, global FSC and histogram, and cryo-EM maps colored by local resolution for the Omicron S-55A8 Fab and Omicron S-55A8/58G6 Fab datasets

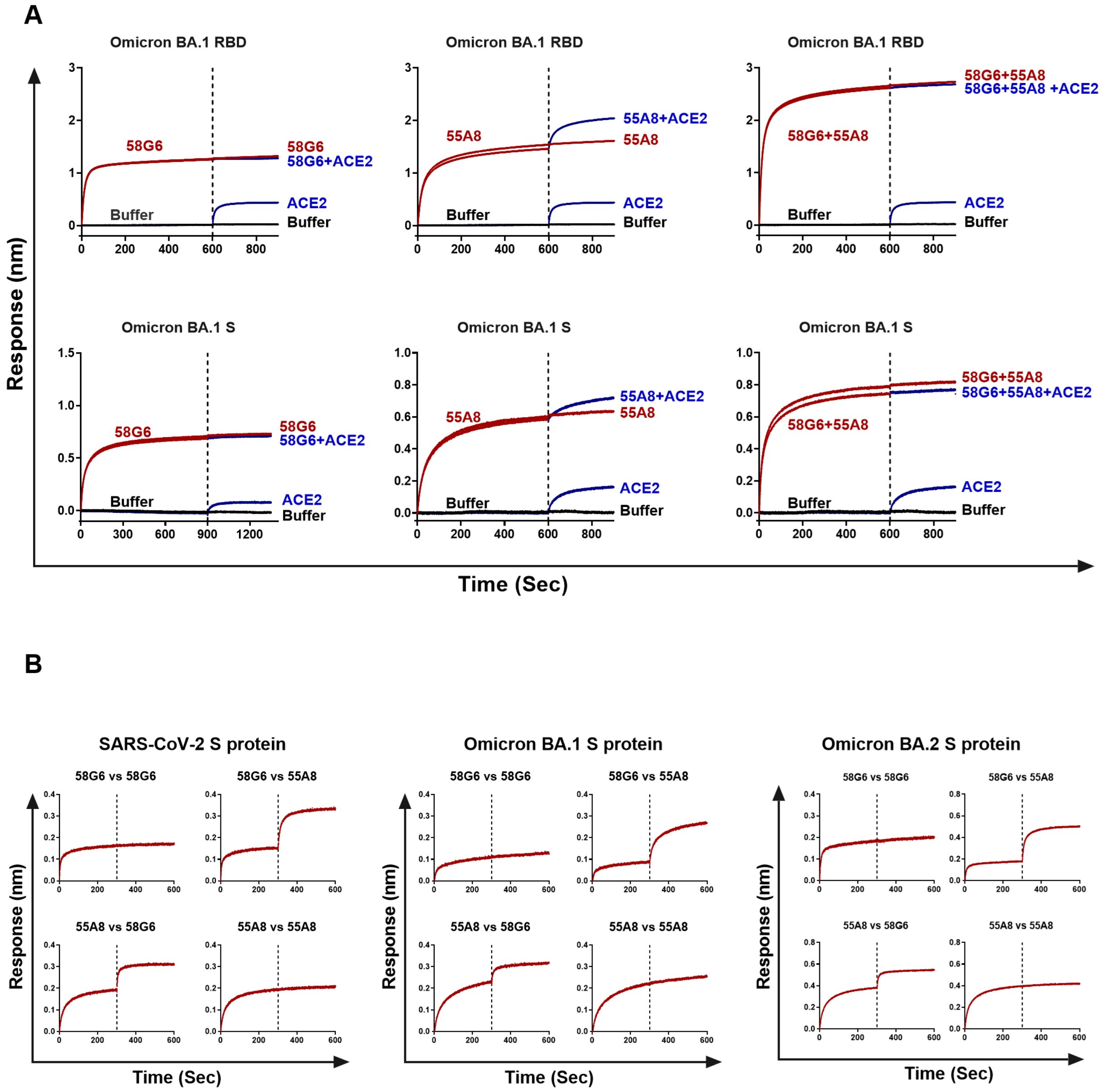

Supplemental Figure 4. Competition experiment by BLI. (**A**) Competition between ACE2 and 58G6, 55A8 or a mixture of 58G6 and 55A8 for binding to the Omicron BA.1 RBD (top) and Omicron BA.1 S protein (down). (**B**) The sequential binding of 58G6 and 55A8 to the SARS-CoV-2, Omicron BA.1 or Omicron BA.2 S protein measured by BLI. Association was measured first for the antibodies indicated on the left, followed by association of the antibodies indicated on the right.

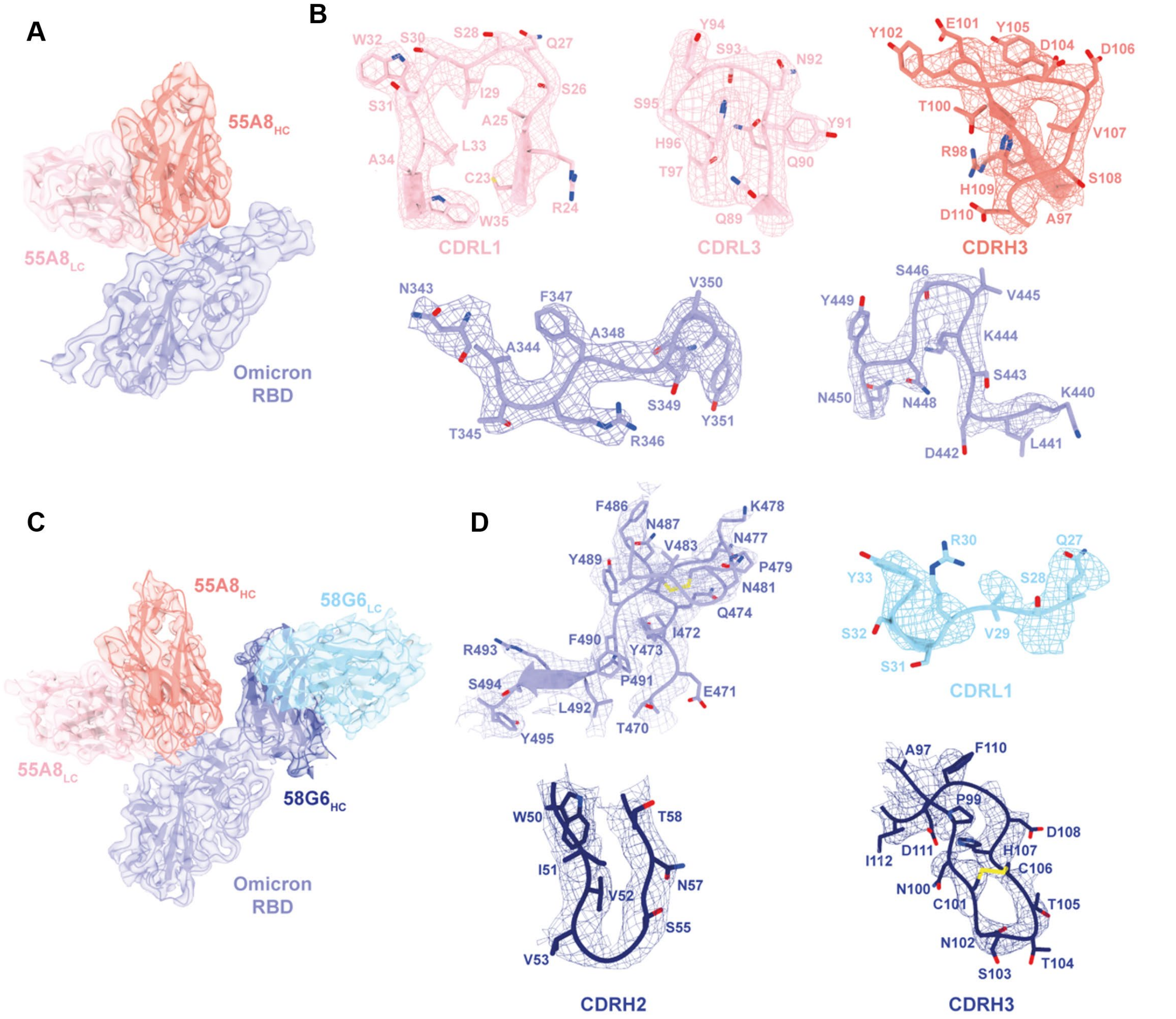

Supplemental Figure 5. Density maps and atomic models. Cryo-EM maps of the binding interface between the Omicron RBD and 55A8 Fab (**A**) or 55A8/58G6 Fab (**C**) variable domains. Density maps (mesh) and related 55A8 CDR (**B**) or 58G6 CDR (**D**) atomic models. Residues are shown as sticks, oxygen atoms are colored red, nitrogen atoms are colored blue, and sulfur atoms are colored yellow.

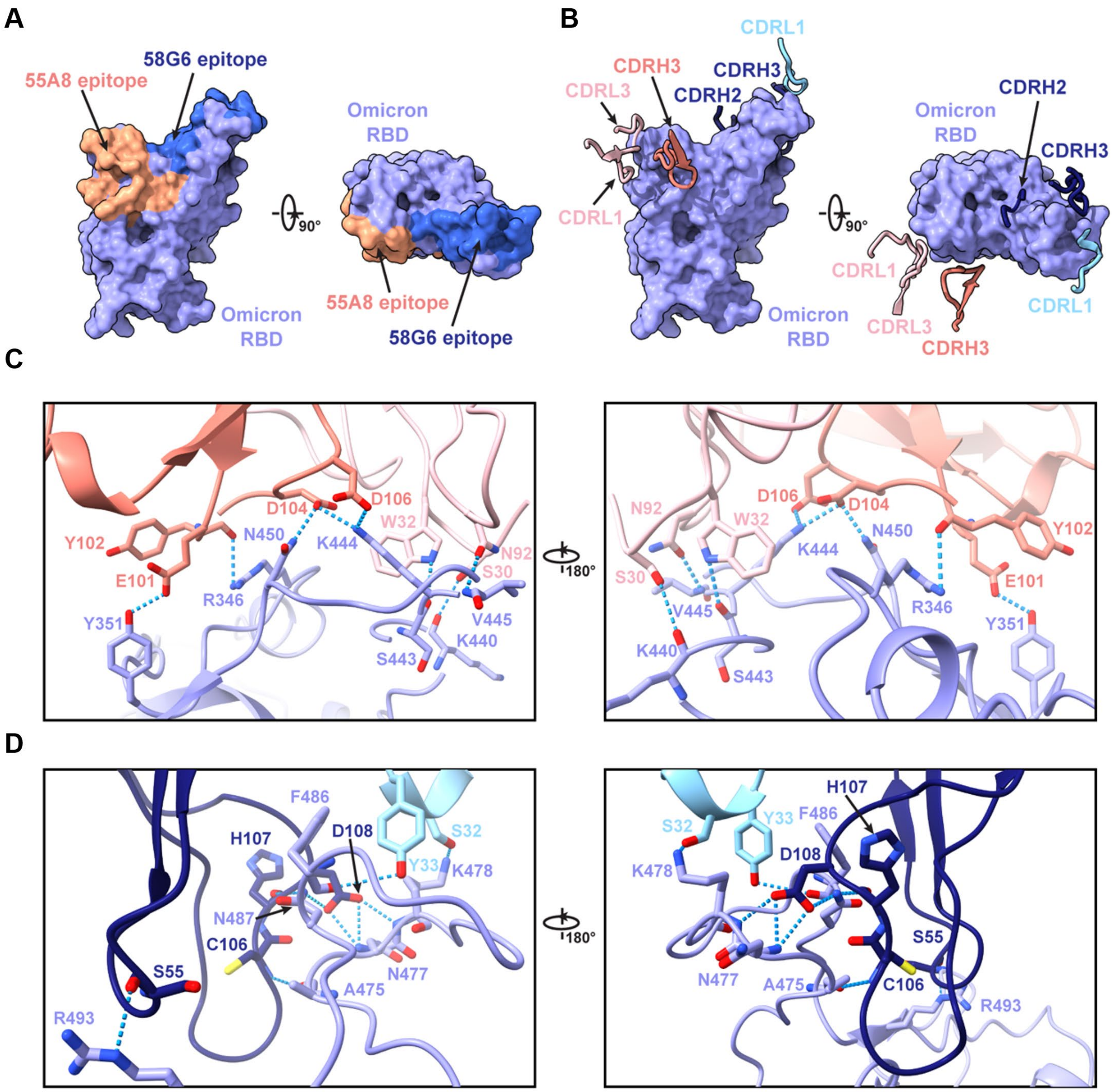

Supplemental Figure 6. Detailed interactions between 55A8/58G6 cocktails and the Omicron BA.1 RBD. (**A**) Surface representation of the 55A8 and 58G6 epitopes on the Omicron RBD surface. (**B**) CDR loops of the 55A8 Fab and 58G6 Fab overlaid on the surface representation of the Omicron RBD. (**C-D**) Potential hydrogen bonds (shown as blue dashed lines) at the binding interface between the Omicron RBD and 55A8 Fab (**C**) or 58G6 Fab (**D**).

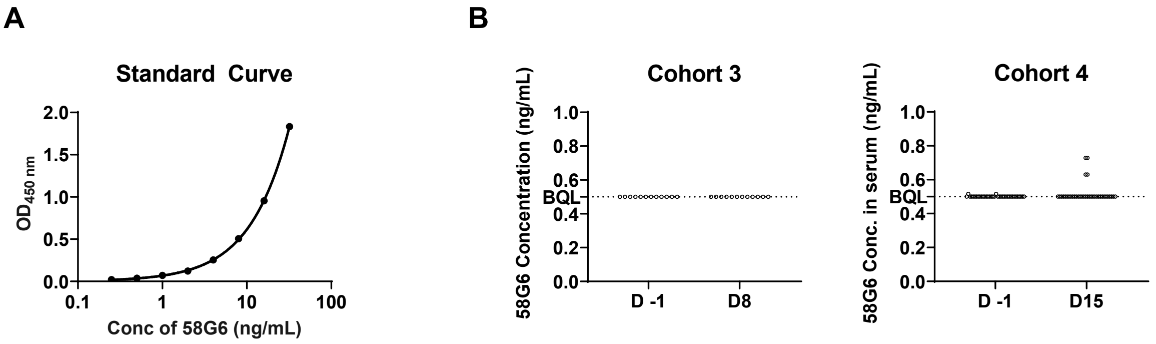

Supplemental Figure 7. Serum concentrations of NAb 58G6 in Cohort 3 and 4 of A8G6 nasal spray trial. (A) Standard ELISA fitting curve. (B) Serum concentrations of NAb 58G6 was measured by a standard ELISA experiment which contain a pair of antibodies that can specifically bind to NAb 58G6. The dashed line of BQL indicates Below the Quantization Limit (0.5 ng/mL). D -1 is the day before dosing; D8 is day 8 post last dosing; D15 is day 15 post last dosing.

Supplemental Table 1: Cryo-EM data collection, model refinement and validation statistics for the Omicron S-55A8 Fab datasets.

	1-up/2-down Class 1	2-up/1-down Class 2	55A8-RBD Local
**Data collection and processing**			
**Voltage (kV)**	300	300	300
**Detector**	K3	K3	K3
**Pixel size (Å)**	0.82	0.82	0.82
**Electron dose (e^-/Å^2)**	60	60	60
**Defocus range**	-1.2 to -2.8	-1.2 to -2.8	-1.2 to -2.8
**Final particles**	82,383	94,894	181,072
**Final resolution (Å)**	3.41	3.39	3.36
**Model refinement**			
**Map-model CC (mask)**	0.86	0.87	0.87
**Initial model used**	7WS5	7WS5	7WS6
**RMSD**			
Bond lengths (Å)	0.003	0.003	0.004
Bond angles (°)	0.571	0.561	0.628
Molprobity score	1.58	1.65	1.66
Clash score	7.60	8.47	6.84
Rotamer outliers (%)	0.00	0.00	0.00
Cβ outliers (%)	0.00	0.00	0.00
CaBLAM outliers (%)	2.41	2.66	2.68
**Ramachandran statistics**			
Favored (%)	97.07	96.82	95.92
Allowed (%)	2.93	3.18	4.08
Outliers (%)	0.00	0.00	0.00

Supplemental Table 2: Cryo-EM data collection, model refinement and validation statistics for the Omicron S-55A8 Fab-58G6 Fab datasets.

	1-up/2-down Class 3	2-up/1-down Class 4	55A8/58G6-RBD Local
**Data collection and processing**			
**Voltage (kV)**	300	300	300
**Detector**	K3	K3	K3
**Pixel size (Å)**	0.82	0.82	0.82
**Electron dose (e^-/Å^2)**	60	60	60
**Defocus range**	-1.2 to -2.8	-1.2 to -2.8	-1.2 to -2.8
**Final particles**	216,663	204,153	354,108
**Final resolution (Å)**	3.29	3.30	3.27
**Model refinement**			
**Map-model CC (mask)**	0.84	0.88	0.82
**Initial model used**	7WS5	7WS5	7WS6
**RMSD**			
Bond lengths (Å)	0.003	0.004	0.003
Bond angles (°)	0.581	0.551	0.563
Molprobity score	1.64	1.56	1.78
Clash score	8.72	6.79	7.60
Rotamer outliers (%)	0.00	0.00	0.00
Cβ outliers (%)	0.00	0.00	0.00
CaBLAM outliers (%)	2.40	2.40	2.69
**Ramachandran statistics**			
Favored (%)	96.99	96.91	94.71
Allowed (%)	3.01	3.09	5.29
Outliers (%)	0.00	0.00	0.00

Supplemental Table 3 Composition and Concentration of Excipients Used in A8G6 Nasal Spray Formulation

**Excipients**	**Concentration/mL**
$Na_2HPO_4 \cdot 7H_2O$	0.50 mg
$Na_2HPO_4 \cdot H_2O$	2.52 mg
D-(+)-Trehalose dihydrate	44.21 mg
Glycerol	20.00 mg
Hydroxypropyl methylcellulose (HPMC)	0.20 mg
Tween 80	0.10 mg
Benzalkonium chloride	0.10 mg
Injection Grade Water	Dissolve to 1mL

Supplemental Table 4: Baseline participant demographic characteristics.

Project/Indicator	Placebo group	Test group	Total
**Age (years)**			
N(Nmiss)	18(0)	90(0)	108(0)
Mean±SD	25.61±4.17	26.64±6.26	26.47±5.96
Median(Q1,Q3)	25.00(23.00,26.00)	25.00(24.00,26.00)	25.00(24.00,26.00)
Min, Max	19.00,37.00	20.00,52.00	19.00,52.00
<18 years old	0(0.00%)	0(0.00%)	0(0.00%)
≥18, <40 years old	18(100.00%)	85 (94.44%)	103 (95.37%)
≥40, <65 years old	0(0.00%)	5(5.56%)	5(4.63%)
≥65, <75 years old	0(0.00%)	0(0.00%)	0(0.00%)
≥75 years old	0(0.00%)	0(0.00%)	0(0.00%)
Total	18(100.00%)	90(100.00%)	108(100.00%)
**Gender [n(%)]**			
Female	15 (83.33%)	58 (64.44%)	73 (67.59%)
Male	3 (16.67%)	32 (35.56%)	35 (32.41%)
Total	18(100.00%)	90(100.00%)	108(100.00%)
**Ethnicity [n(%)]**			
Han Chinese	14 (77.78%)	77 (85.56%)	91 (84.26%)
Others	4 (22.22%)	13 (14.44%)	17 (15.74%)
Total	18(100.00%)	90(100.00%)	108(100.00%)
**Height (cm)**			
N(Nmiss)	18(0)	90(0)	108(0)
Mean±SD	160.67±5.94	164.86±8.07	164.16±7.89
Median(Q1,Q3)	160.00(156.00,163.00)	163.00(158.00,170.00)	163.00(158.00,170.00)
Min,Max	152.00,174.00	151.00,183.00	151.00,183.00
**Body weight (kg)**			
N(Nmiss)	18(0)	90(0)	108(0)
Mean±SD	54.67±7.41	58.79±11.64	58.10±11.13
Median(Q1,Q3)	54.00(49.00,60.00)	55.00(50.00,65.00)	55.00(50.00,65.00)
Min,Max	46.00,75.00	43.00,95.00	43.00,95.00
**BMI(kg/m)^2)**			
N(Nmiss)	18(0)	90(0)	108(0)
Mean±SD	21.12±1.82	21.48±2.92	21.42±2.76
Median(Q1,Q3)	21.00(20.40,21.50)	20.75(19.60,22.90)	20.80(19.70,22.80)
Min,Max	18.00,24.80	16.30,29.70	16.30,29.70

Supplemental Table 5: Summary of adverse effects in all subjects.

	Placebo group			Test group			Total		
	Example	Number of cases	Incidence %	Example	Number of cases	Incidence %	Example	Number of cases	Incidence %
Adverse events	8	2	11.11	6	6	6.67	14	8	7.41
Adverse reactions	0	0	0	6	6	6.67	6	6	5.56
Serious adverse events	0	0	0	0	0	0	0	0	0
Serious adverse reactions	0	0	0	0	0	0	0	0	0
**Grading adverse events**									
Grade I	7	1	5.56	6	6	6.67	13	7	6.48
Grade II	1	1	5.56	0	0	0	1	1	0.93
Grade III	0	0	0	0	0	0	0	0	0
Grade IV	0	0	0	0	0	0	0	0	0
Grade V	0	0	0	0	0	0	0	0	0
**Relationship to study drug**									
Definitely related	0	0	0	0	0	0	0	0	0
probably related	0	0	0	0	0	0	0	0	0
Possibly related	0	0	0	6	6	6.67	6	6	5.56
Possibly unrelated	8	2	11.11	0	0	0	8	2	1.85
To be evaluated	0	0	0	0	0	0	0	0	0
Unable to evaluate	0	0	0	0	0	0	0	0	0
**Adverse events leading to shedding**	0	0	0	0	0	0	0	0	0
**Adverse reactions leading to shedding**	0	0	0	0	0	0	0	0	0
Unable to evaluate	0	0	0	0	0	0	0	0	0
**Adverse events leading to shedding**	0	0	0	0	0	0	0	0	0
**Adverse reactions leading to shedding**	0	0	0	0	0	0	0	0	0

Supplemental Table 6: Classification statistics of adverse events.

Whole Body System (SOC)	Placebo group			Test group			Total		
Adverse Events (PT)	Example	Number of cases	Incidence (%)	Example	Number of cases	Incidence (%)	Example	Number of cases	Incidence (%)
**Various types of inspections**	3	1	5.56	0	0	0	3	1	0.93
White blood cell count increased	1	1	5.56	0	0	0	1	1	0.93
Lymphocyte count decreased	1	1	5.56	0	0	0	1	1	0.93
Neutrophil count increased	1	1	5.56	0	0	0	1	1	0.93
**All kinds of neurological diseases**	1	1	5.56	0	0	0	1	1	0.93
Dizziness	1	1	5.56	0	0	0	1	1	0.93
**Respiratory, thoracic and mediastinal diseases**	1	1	5.56	6	6	6.67	7	7	6.48
Epistaxis	1	1	5.56	4	4	4.44	5	5	4.63
Runny nose	0	0	0	2	2	2.22	2	2	1.85
**Gastrointestinal system diseases**	3	1	5.56	0	0	0	3	1	0.93
Abdominal pain	1	1	5.56	0	0	0	1	1	0.93
Diarrhea	1	1	5.56	0	0	0	1	1	0.93
Vomiting	1	1	5.56	0	0	0	1	1	0.93
